# Shear stress unveils patient-specific transcriptional signatures in PAH: Towards personalized molecular diagnostics

**DOI:** 10.7150/thno.105729

**Published:** 2025-01-02

**Authors:** Corey Wittig, Jakob M König, Xiaoke Pan, Jurjan Aman, Harm-Jan Bogaard, Paul B Yu, Wolfgang M Kuebler, Katharina Baum, Robert Szulcek

**Affiliations:** 1 Laboratory of in vitro modelling systems of pulmonary and thrombotic diseases, Institute of Physiology, Charité-Universitätsmedizin Berlin, corporate member of Freie Universitätsmedizin Berlin and Humboldt-Universität zu Berlin, Charitéplatz 1, 10117 Berlin, Germany.; 2 DZHK (German Centre for Cardiovascular Research), partner site Berlin, Berlin, Germany.; 3 DZL (German Centre for Lung Research), partner site Berlin, Berlin, Germany.; 4 Institute of Physiology, Charité-Universitätsmedizin Berlin, corporate member of Freie Universitätsmedizin Berlin and Humboldt-Universität zu Berlin, Charitéplatz 1, 10117 Berlin, Germany.; 5 Department of Pulmonary Medicine, Amsterdam UMC, VU University Medical Center, Amsterdam Cardiovascular Sciences, Amsterdam, The Netherlands.; 6 Cardiovascular Research Center, Division of Cardiovascular Medicine, Department of Medicine, Massachusetts General Hospital, Harvard Medical School, Boston, MA, USA.; 7 Departments of Physiology and Surgery, University of Toronto, Toronto, ON, Canada.; 8 Department of Mathematics and Computer Science, Free University Berlin, 14195 Berlin, Germany.; 9 Hasso Plattner Institute, Digital Engineering Faculty, University of Potsdam, 14482 Potsdam, Germany.; 10 Windreich Department of Artificial Intelligence and Human Health & Hasso Plattner Institute for Digital Health at Mount Sinai, Icahn School of Medicine at Mount Sinai, 10029 New York City, NY, USA.; 11 Deutsches Herzzentrum der Charité, Department of Cardiac Anesthesiology and Intensive Care Medicine, Augustenburger Platz 1, 13353 Berlin, Germany.

**Keywords:** molecular profiling, transcriptomics, heterogeneity, shear stress, pulmonary arterial hypertension

## Abstract

**Rationale:** Pulmonary arterial hypertension (PAH) is a life-threatening disorder characterized by increased pulmonary blood pressures and regional inhomogeneities in flows, with diagnostic and treatment challenges arising from diverse underlying pathogenic mechanisms. Conventional *in vitro* models often obscure the mechanistic nuances of PAH by failing to replicate the dynamic mechanical environment of the diseased lung, limiting the identification of specific molecular patterns. To address this, we employed an *in vitro* shear stress model simulating physiological or pathological conditions to explore the transcriptional heterogeneity of human pulmonary microvascular endothelial cells (hPMECs) from PAH patients and healthy controls within their respective biomechanical context.

**Methods & Results:** hPMECs from PAH patients and controls were exposed to static, low shear stress (LSS), and high shear stress (HSS) conditions, followed by bulk RNA-sequencing. While increasing shear stress resulted in a greater number of differentially expressed genes, traditional grouped analysis showed minimal overall transcriptional differences. Further, pathway enrichment analysis indicated common shear-induced responses in both groups, suggesting that standard analysis methods may mask meaningful disease-specific changes.

Crucially, detailed dimensionality reduction analyses revealed pronounced inter-patient variability among PAH donors in response to increasing shear stress, facilitating the identification of 398 genes driving this transcriptional heterogeneity. Unsupervised clustering of these high-variability genes enabled the sub-classification of patients based on their unique transcriptomic profiles, each linked to specific combinations of PAH associated pathogenic pathways such as mesenchymal transition, inflammation, metabolism, extracellular matrix remodeling, and cell cycle/DNA damage signaling. Importantly, re-analysis of published peripheral blood mononuclear cell (PBMC) omics data from PAH patients confirmed the clinical feasibility to utilize these high-variability genes as a non-invasive, accessible approach for molecular patient stratification.

**Conclusion:** Our study uncovers patient-specific transcriptomic patterns in PAH, providing a novel molecular sub-classification strategy. These findings represent a significant step toward personalized molecular diagnostics in PAH and eventual therapeutic interventions for clinically well-defined PAH patients, with potential applications in clinically accessible cell populations such as PBMCs.

## Introduction

Pulmonary arterial hypertension (PAH) is a life-threatening disorder characterized by increased pulmonary vascular resistance, elevated pulmonary arterial pressure, and ultimately, right ventricular failure 1. Despite advancements in understanding the clinical manifestations of PAH, its underlying pathogenic mechanisms remain highly heterogeneous 2. This heterogeneity has presented a major challenge for both diagnosis and treatment, since growing clinical trial cohorts and registries have failed to identify a common molecular denominator across patients 3-6. As a result, PAH remains a lethal condition where generalized therapeutic options often fail to capture patient-specific disease mechanisms and even lead to serious adverse events in some 7-9. This underscores the critical need for personalized diagnostic and therapeutic strategies. Thus, innovative personalized research approaches are crucial for overcoming the challenge of molecular heterogeneity and improving patient outcomes in PAH.

Central to the complexity of PAH is the role of biomechanical forces, particularly blood flow-induced shear stress resulting from the narrowing of the pulmonary vasculature 10. While shear stress, the tangential force generated by blood flow, is necessary for normal endothelial cell function 11, supra-physiological shear stresses have long been implicated in PAH progression 12, 13. Specifically, the shear-associated repetitive mechanical damage to the vascular wall and consequent emergence of pheno- and genotypically altered cells have been proposed to be a central contributor to the obliterative remodeling and formation of typical plexogenic lesions in PAH 14. However, its precise role - whether as a disease inducer, maintenance factor, or exacerbator - remains unclear. Interestingly, despite its recognized importance, the molecular impact of both physiological and pathological shear stress on PAH lung endothelial cells is not fully understood.

Conventional *in vitro* models for PAH typically rely on static culture systems, which fail to replicate the dynamic and spatially heterogenous biomechanical forces present in the pulmonary vasculature of PAH patients. These models may obscure key transcriptional differences between healthy and diseased cells, limiting the discovery of specific molecular pathways. Our previous work identified a significant delay in the morphological adaptation of human pulmonary microvascular endothelial cells (hPMECs) from PAH patients to high shear stress 15. These findings point to an underlying mechanotransduction defect in hPMECs that may contribute to the disease. However, the transcriptional changes in the shear exposed PAH hPMECs remained unclear.

To address this, we utilized our *in vitro* shear stress model that simulates physiological and pathological shear stress conditions, allowing for the investigation of shear-induced transcriptional changes in hPMECs from PAH patients and healthy controls by bulk RNA-sequencing.

We hypothesized that increasing shear stress would unveil distinct patient-specific transcriptional signatures in hPMECs, reflective of individual molecular mechanisms contributing to PAH. These signatures hold potential for informing personalized molecular diagnostics, advancing the field toward more individualized therapeutic interventions.

## Methods

### Primary cell isolation

Control hPMECs (n=4: 3 male, 1 female) were isolated from pulmonary lobectomies for suspected or proven non-small cell lung carcinomas, while PAH hPMECs (n=4: 3 female, 1 male) were isolated from peripheral microvascular tissue of patients with clinically well-characterized Group 1 PAH (Table 1). Cell isolation was based on a previously-published protocol, and cells showed typical growth patterns that formed cobblestone monolayers and were positive for endothelial markers 15. The tissue collection and cell isolation were approved by the institutional review board of the VU University Medical Center (VUmc, Amsterdam, the Netherlands, protocol-nr: 2012/306), and written, informed consent was obtained from all participants or their surrogates.

### Cell culture and fluid flow techniques

hPMECs were cultured in Endothelial Cell Medium with 5% FCS, 1% Penicillin-Streptomycin, 1% Endothelial Cell Growth Supplement (ScienCell, #1001), and additional 1% Non-Essential Amino Acids (Gibco, #11140-035). All cells used were passage 3-5. Single-channel ibidi µ-Slides I Luer 0.4 (ibidi, #80176) were seeded with 40,000 cells/cm^2^ and cells were allowed to attach overnight (four channels per donor). Each channel was then subjected to one of three fluid flow conditions for 24 hours: no flow (“Static”), 2.5 dyn/cm^2^ physiological low (“LSS”), or 15 dyn/cm^2^ supra-physiological high unidirectional shear stress (“HSS”). Following this, the cells were immediately collected in QIAzol Lysis Reagent (Qiagen, #79306) for bulk RNA-seq analysis and quantitative real-time polymerase chain reaction (qPCR) validation. RNA was isolated with the miRNeasy Micro Kit (Qiagen, #217084).

### RNA purification

Total RNA was purified with the MagMAX-96 for Microarrays Total RNA Isolation kit (ThermoFisher, AM1839) according to the manufacturer's instructions, in which genomic DNA was removed using MagMAXTurboDNase buffer and TURBO DNase. mRNA was purified from total RNA using Dynabeads mRNA purification kit (Invitrogen, #61006) according to the manufacturer's instructions.

### Library preparation and RNA-sequencing

ScriptSeq mRNA-Seq Library Preparation Kit (Epicentre, SS10906) was used to prepare strand-specific RNA-sequencing libraries. Twelve-cycle polymerase chain reaction was performed to amplify libraries. Sequencing was performed on Illumina HiSeq2000 by a 33-cycle multiplexed, single-read run. Raw sequence data (BCL-files) were converted to FASTQ format via Illumina Casava 1.8.2. Reads were decoded based on their barcodes and read quality was evaluated using FastQC 16. Reads were mapped to the human transcriptome (hg38) and reads mapping to sense strand exons were summed at the gene level using ArrayStudio (OmicSoft).

### RNA-seq data preparation and filtering

Gene read counts were analyzed using the ExpressAnalyst platform 17. Data filtering was implemented to exclude likely uninformative or erroneous data by removing unannotated genes, the bottom 4% low abundance genes, and the bottom 15% low variance genes, assessed over all measured samples. Disease group (control, PAH) served as the primary factor, with flow condition (Static, LSS, HSS) as the secondary factor. Patient pairing was not performed due to platform limitations (max. 2 factors).

### Differential expression and over-representation analysis

For differential expression/over-representation analysis, gene read counts were processed utilizing the DESeq2 method 18 within the ExpressAnalyst platform 17 to determine differentially-expressed genes (DEGs) across all possible comparisons. Over-representation analysis was subsequently conducted for each pairwise comparison's DEGs against the full filtered dataset background using the KEGG database.

### Similarity analysis in reduced-dimensionality data

For similarity analysis, gene read counts were normalized sample-wise as log_2_(counts per million [CPM]), then full filtered expression data was subjected to three-component canonical correlation analysis (CCA) on each control *vs.* PAH flow condition pair using the *sklearn 1.1.1* Python package (*e.g.*, control Static expressions *vs.* PAH Static expressions). Similarity between control and PAH at defined flow conditions was assessed by the CCA correlation coefficient between the two data sets. A higher correlation coefficient indicates a stronger alignment of gene expression patterns, indicating greater similarity.

### Grouping and spatial metrics analysis in reduced-dimensionality data

For condition grouping and spatial metrics analysis, gene read counts were normalized sample-wise as log_2_(CPM), then subjected to Principal Component Analysis (PCA) using the ExpressAnalyst platform 17, where we considered the first three components to plot each sample in 3-dimensional reduced space and visualize their distributions. Ellipsoids representing 95% confidence intervals were delineated around groups in the 3D PCA space for visualization and to characterize separation between groups and heterogeneity within groups.

### Gene variability analysis

For gene variability analysis, gene read counts were normalized sample-wise as counts per million. The coefficient of variation (CV, Equation 1) was then calculated on the normalized data for each individual gene within each unique group, applying a small sample size bias correction factor of (1+1/4n), where n is the sample size, σ_x_ is sample standard deviation, and x̄ is sample mean 19.

To elucidate the impact of supra-physiological shear stress on variability, ∆CV (CV_HSS_ - CV_Static_) was calculated for each gene per disease group. Only genes that had read counts >0 in all samples within the considered flow conditions were kept for further investigation. To identify highly variable genes in PAH responses to shear stress that are not highly variable in control shear responses, ∆∆CV between ∆CV_Control_ and ∆CV_PAH_ was calculated (Equation 2). Genes with a cut-off of >50% ∆∆CV were used for later inputs as “top variable genes” in PAH shear stress responses.

Equation 1) CV (%) = (1 + 1/4n) * (σ_x_/x̄) * 100

Equation 2) ∆∆CV = ∆CV_PAH,Static→HSS_ - ∆CV_Control,Static→HSS_

### Individual patient analysis

For individual patient analysis, gene read counts were normalized sample-wise as log_2_(CPM). Z-scores were calculated to normalize each gene separately over the PAH samples, using the “top variable genes” log_2_CPM data from PAH hPMECs under HSS, chosen for its high inter-patient variability. This process aimed to develop a relative PAH patient-to-patient signature based on inter-patient heterogeneity as a readout for dysregulation in at least one patient. Z-scored “top variable genes” were subjected to unsupervised Partitioning Around Medoids (PAM) clustering to identify patient-specific gene clusters. These gene clusters were then used for over-representation analysis against the full filtered dataset background, utilizing the Reactome, KEGG, and MSigDB databases. Additionally, the average log_2_(fold change [FC]) for the “top variable genes” in each significantly enriched pathway was calculated individually for each PAH patient compared to the full control group. This analysis provided insights into how the relative PAH-PAH differences scale in an individual PAH patient relative to the control group.

### qPCR validation of RNA-seq data

Purified RNA from hPMECs was used to synthesize cDNA using iScript cDNA Synthesis Kit (Bio-Rad, #1708890). qPCR was performed with iQ SYBR Green Supermix (Bio-Rad, #1708880) and run in triplicate using the Bio-Rad CFX384 Real-Time PCR Detection System. Analysis of qPCR results was performed using relative expression for ∆∆CV calculation, and the ∆∆Ct method for determining fold changes. *RPLP0* or *RPL27* were used as established non-shear-responsive internal controls. Primer efficiencies and melt temperatures were tested on general cDNA prior to usage, and amplification specificity was ensured through post-amplification melting curve analysis. Primer sequences are listed in Table S1.

### Selection and analysis of published PAH PBMC datasets for validation

NCBI's Gene Expression Omnibus (GEO) was queried for all publicly available RNA-seq or microarray data on PAH peripheral blood mononuclear cells (PBMCs), of which three microarray datasets were identified 20-22. We selected the dataset with the largest cohort (n=41 control, n=30 iPAH) for further analysis and validation of our findings (GEO accession: GSE33463) 21. As stated in the GEO dataset description, the dataset consists of scaled microarray expression values for all probes in each sample to a median of 256, followed by log_2_-transformation. For our analysis, we used the control and PAH patient data, and if a gene was represented by multiple probes, the probe with the highest expression value was chosen for further investigation. To align with our previous analysis of hPMECs, we then calculated the average log_2_FC in accordance to our previously identified “top variable genes”, comparing each PAH patient individually to the full control group.

### Statistics

P-values were calculated in using the hypergeometric test (one-tailed Fisher's exact test) across all analyses except the DESeq2-based grouped analysis, where p-values were calculated with the Wald test. Correction for multiple hypotheses testing was consistently performed using the Benjamini-Hochberg method (False Discovery Rate procedure), and significance was established with an adjusted p-value ≤ 0.05 (* p ≤ 0.05, ** p ≤ 0.01, *** p ≤ 0.001). Additionally, pathway enrichments were deemed significant if there were at least three DEGs in the gene set. Figures were generated using GraphPad Prism 7.0a (GraphPad Software, Boston, Massachusetts) unless otherwise stated.

## Results

hPMECs derived from control donors and PAH patients were exposed to differing unidirectional fluid flow conditions (either “Static” 0 dyn/cm^2^, “LSS” 2.5 dyn/cm^2^, or “HSS” 15 dyn/cm^2^) for 24 hours to model *in vitro* no-flow, physiological, and supra-physiological high fluid shear stress in line with previously published protocols 23, 24. hPMECs were confirmed to have sensed varying degrees of shear stress through qPCR-measured expression changes in the shear-responsive transcriptional master regulator *KLF2*, changes in multiple shear-sensitive genes in the RNA-seq dataset, and morphological adaptation (Figure S1).

To discern how these mechano-environmental changes might impact PAH transcriptomic profiles *in vitro*, we followed a comprehensive analytical approach as detailed in Figure S2 and the Methods section. Overall, 26,479 genes were present in the dataset, reduced to 24,603 by removal of unmatched and unannotated genes, and finally condensed to the working dataset of 14,493 genes by filtering out low abundance and low variability genes (Figure S3).

### Despite a shear dependent increase in differential gene expression, no pathway enrichments were identified in PAH hPMECs

**Figure 1:**
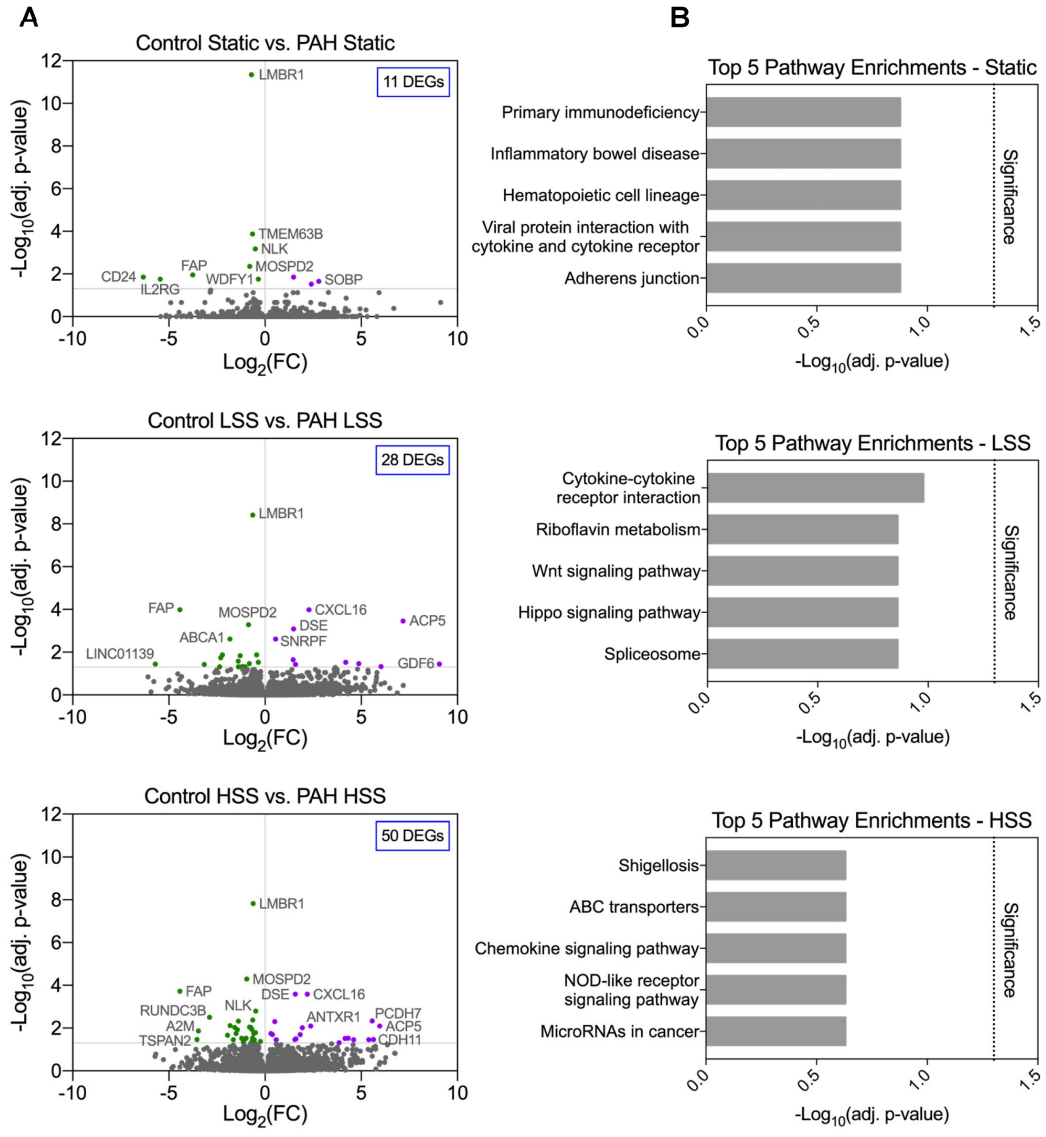
** Minimal significant differences are found in PAH hPMECs within each flow condition by grouped analysis. (A)** Grouped comparison of control and PAH hPMECs within distinct flow conditions. Under static conditions, 11 genes were differentially expressed (7 unique to static). Under low shear stress conditions, 28 genes were differentially expressed (14 unique to LSS). Under high shear stress conditions, 50 genes were differentially expressed (36 unique to HSS). **(B)** Top 5 enriched pathways in each comparison based on over-representation analysis. No significant pathway enrichments were identified.

PAH and control hPMECs gene expression profiles within each flow condition were compared (*i.e.,* Static *vs.* Static, LSS *vs.* LSS, HSS *vs.* HSS) to explore if PAH and control hPMECs present a statistically differentiable profile under static conditions or after shear stress exposure, and whether HSS exacerbates pathway dysregulations in PAH hPMECs. Differential gene expression analysis identified 11 DEGs (7 unique) in the “Static” comparison, 28 DEGs (14 unique) in the “LSS” comparison, and 50 DEGs (36 unique) in the “HSS” comparison (Figure 1A, Table S2). Despite the increase in DEGs with increasing shear stress, no significant pathway enrichments distinguishing PAH from control hPMECs were found across all conditions (Figure 1B).

### Overall responses to fluid flow are conserved in PAH hPMECs despite transcriptional differences between flow conditions in PAH

**Figure 2:**
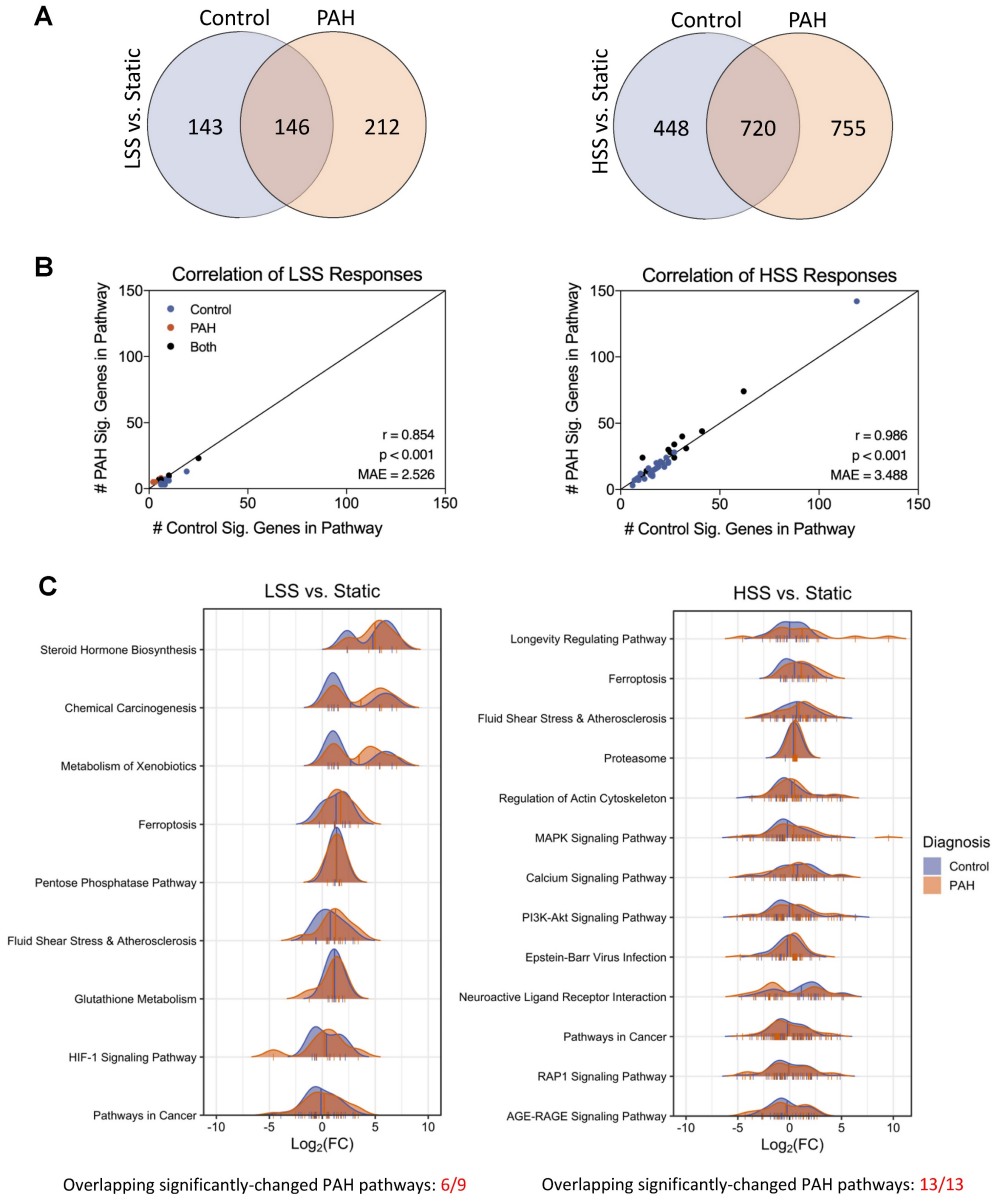
** Overall responses to fluid flow are conserved in PAH hPMECs despite transcriptional differences between flow conditions.** (A) Quantification of DEGs for LSS *vs.* Static and HSS *vs.* Static in both PAH and control groups, with overlaps noted. The number of non-overlapping DEGs between PAH and controls increased from 355 non-overlapping DEGs at LSS *vs.* Static, to 1203 non-overlapping DEGs at HSS *vs.* Static. (B) KEGG pathway enrichment analysis for both disease condition groups in LSS *vs.* Static and HSS *vs.* Static. In LSS *vs.* Static, 9 pathways were significantly changed for PAH and 16 for control (6 overlapping). In HSS *vs.* Static, 13 pathways were significantly changed for PAH and 41 for control (13 overlapping). Including all significantly changed pathways in either control or PAH, the correlation in the number of DEGs per pathway between control and PAH was very strong in both LSS *vs.* Static and HSS *vs.* Static, indicating minimal differences between PAH and control flow responses. (C) Density plots for each significantly enriched pathway in PAH flow responses, where the curve represents the expression change profile in terms of log_2_FC of each DEG in the pathway (marked as ticks on pathway x-axes). Mean pathway log_2_FC are marked with a vertical line. Figure generated using the *ggridges 0.5.6* R package.

To investigate how hPMECs from PAH patients and controls respond to changing flow conditions, we then examined the following condition sets: PAH LSS *vs.* PAH Static, control LSS *vs.* control Static, PAH HSS *vs.* PAH Static, control HSS *vs.* control Static. We found that the number of non-overlapping DEGs between PAH and control flow responses increased substantially as the shear stress intensified. Specifically, there were 355 non-overlapping DEGs between PAH and controls at LSS *vs.* Static, which increased to 1203 in the HSS *vs.* Static comparison (Figure 2A). These results indicate that HSS amplifies the number of unique differences between control and PAH.

Considering the increased non-overlapping DEGs due to HSS responses, we investigated whether there were also non-overlapping enriched pathways. For LSS *vs.* Static, 9 pathways were significantly changed in PAH and 16 in controls (6 overlapping). For HSS *vs.* Static, 13 pathways were significantly changed in PAH and 41 in controls (13 overlapping). However, considering each pathway significant in control, PAH, or both, the significant DEGs in the pathway gene sets were consistently similar between control and PAH despite the minimal statistical overlap. This was supported by the very strong correlation in the number of significant DEGs in each pathway between control and PAH. Specifically, the correlation was very strong in LSS *vs.* Static (p < 0.001, Pearson r = 0.854, Mean Absolute Error [MAE] = 2.526) and HSS *vs.* Static (p < 0.001, Pearson r = 0.986, MAE = 3.488), where each point is a pathway that is significantly changed in either PAH or control due to shear stress. Here, x is the number of significantly changed genes in that pathway for control, and y is the number of significantly changed genes in that same pathway for PAH (Figure 2B). These results indicate PAH responses remained consistent with control responses, and the lower number of significantly changed pathways in PAH HSS conditions is most likely due to the larger DEG set in PAH increasing the background noise and reducing statistical power.

For this reason, we more deeply investigated the pathways significantly changed in PAH by comparing the fold changes of DEGs associated with these pathways in both PAH and control hPMECs using density plots (Figure 2C, Table S3). This revealed the contour and magnitude of change in significantly changed PAH pathways closely parallels control hPMEC changes, with similar patterns in both groups. The same was observed in density plots of pathways significantly enriched only in control hPMEC flow responses, where PAH hPMEC contours and magnitudes of change closely mirrored controls despite lacking statistical significance (Figure S4). Despite the notable increase in DEG differences between control and PAH with elevated shear stress, these results indicate that overall functional responses to fluid flow are conserved in PAH hPMECs, suggesting that standard enrichment analysis methods may mask meaningful disease-specific changes.

### Dimensionality reduction reveals exacerbation of a distinct PAH phenotype with increasing shear stress, accompanied by increased inter-patient variability

Given the considerable lack of DEG overlap in flow responses of PAH and control hPMECs, we aimed to understand why no significant pathway enrichment differences were found. We therefore investigated the molecular differences between PAH and control hPMECs more deeply, using Canonical Correlation Analysis (CCA) to assess the similarity between gene expression profiles under matching flow conditions (*e.g.*, static control *vs.* static PAH). CCA correlation coefficients revealed that as shear stress increased, the gene expression profiles between control and PAH became progressively dissimilar (Figure S5).

**Figure 3:**
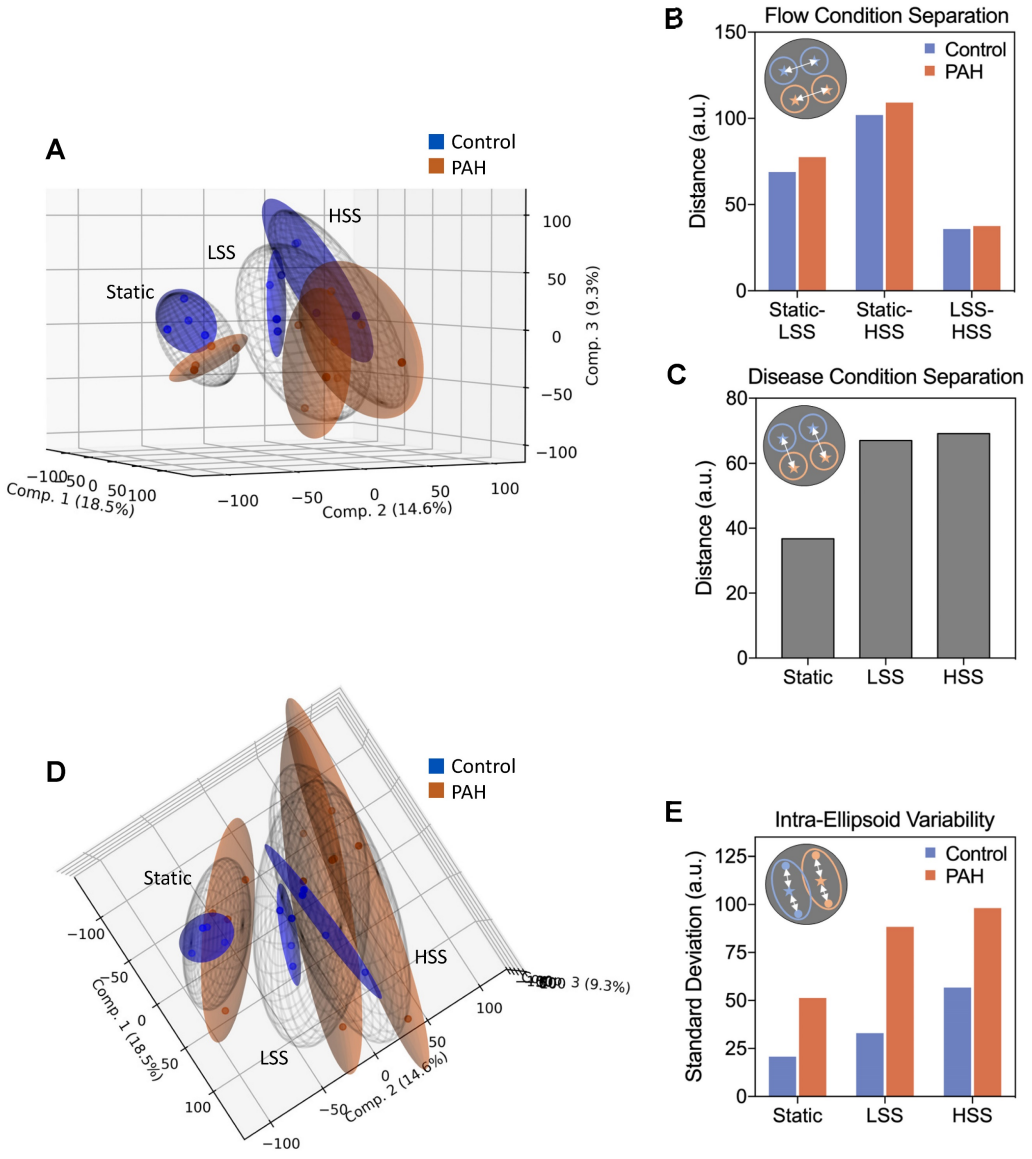
** Dimensionality reduction reveals exacerbation of a distinct PAH phenotype with increasing shear stress, accompanied by increased inter-patient variability.** (A) Principal component analysis (PCA) was performed on the full filtered transcriptome, considering the first three components to reduce each sample into a point in 3D space. 95% confidence ellipsoids were fit to the samples in each condition group, emphasizing group differences. Figure generated using the *matplotlib 3.5.2* Python package. (B) Euclidean distance between the centroids of each flow condition ellipsoid quantifies overall transcriptional shifts due to flow. Minimal differences between control and PAH were observed. (C) Euclidean distance between the centroids of control and PAH within each flow condition quantifies PAH phenotype emergence. Fluid flow was found to drive separation between control and PAH overall expression profiles. (D) Shifting the projection of the PCA 90˚ emphasizes ellipsoid size, correlating directly to inter-sample variability. (E) Intra-ellipsoid variability, measured as standard deviation from the centroid for each ellipsoid, was higher for all PAH *vs.* control comparisons, peaking in PAH HSS.

To further explore this divergence, we employed Principal Component Analysis (PCA), considering the first three components to visualize individual sample distributions in three-dimensional space (Figure 3A). The 95% confidence ellipsoids for each condition revealed clear separations between different flow conditions for both groups, where the distance between flow conditions was generally similar for both PAH and control, mirroring our earlier finding that overall shear responses were conserved in PAH hPMECs (Figure 3B). However, fluid flow considerably increased divergence between control and PAH, underpinning that flow amplifies transcriptomic differences in PAH hPMECs (Figure 3C). Considering the *BMPR2* mutation in PAH02, a known pre-disposition to the development of PAH, we further explored the flow responses of BMP and TGF-beta pathway genes (Figure S6). We found that HSS notably resulted in downregulation of the BMP pathway specifically in PAH02 compared to the other patient samples and controls, demonstrating that shear stress exacerbates underlying PAH signaling.

Lastly, shifting the projection of the PCA 90˚ revealed PAH ellipsoids are substantially larger than their control counterparts, indicating much greater dispersion of the individual PAH hPMEC samples (Figure 3D). We therefore calculated the standard deviation from the centroid for each ellipsoid, finding that PAH patient to patient variability was consistently higher than control variability at all flow conditions, peaking at HSS (Figure 3E).

Considering the lack of significant pathway enrichments from our earlier analyses, these findings show that while shear stress amplifies differences between PAH and controls, each individual PAH patient hPMEC sample may diverge from controls in a distinct way. Therefore, rather than seeking a single unifying pathological signature to explain this general divergence under high shear stress, it may be more insightful to investigate the drivers of inter-patient variability in HSS flow responses as indicators of patient-specific dysregulations.

### Gene variability analysis identifies the drivers of heterogeneity in PAH hPMECs due to increasing shear stress

We thus aimed to determine which genes drove the identified PAH heterogeneity through inconsistent adaptations to HSS. Therefore, we calculated the coefficient of variation (CV) for each gene within defined flow conditions and disease groups (*e.g.*, CV for *VEGFA* in static PAH hPMECs) (Equation 1). As expected, distribution of gene CV values skewed higher in PAH HSS compared to other conditions, confirming the increased patient heterogeneity at HSS (Figure S7).

Based on our results underscoring an exacerbated PAH phenotype with increasing shear stress, we focused on genes with the highest ∆∆CV score from this analysis - the genes that exhibited the greatest variability in how they changed from static to HSS in PAH only (Equation 2). These 398 genes, termed “top variable genes”, were considered the strongest drivers of heterogeneity in PAH responses to HSS that did not vary in control responses to HSS. In other words, these genes demonstrated extreme variability in the PAH group exceeding normal biological variability in controls. Cut-off curation of these genes is visualized in Figure S8.

**Figure 4:**
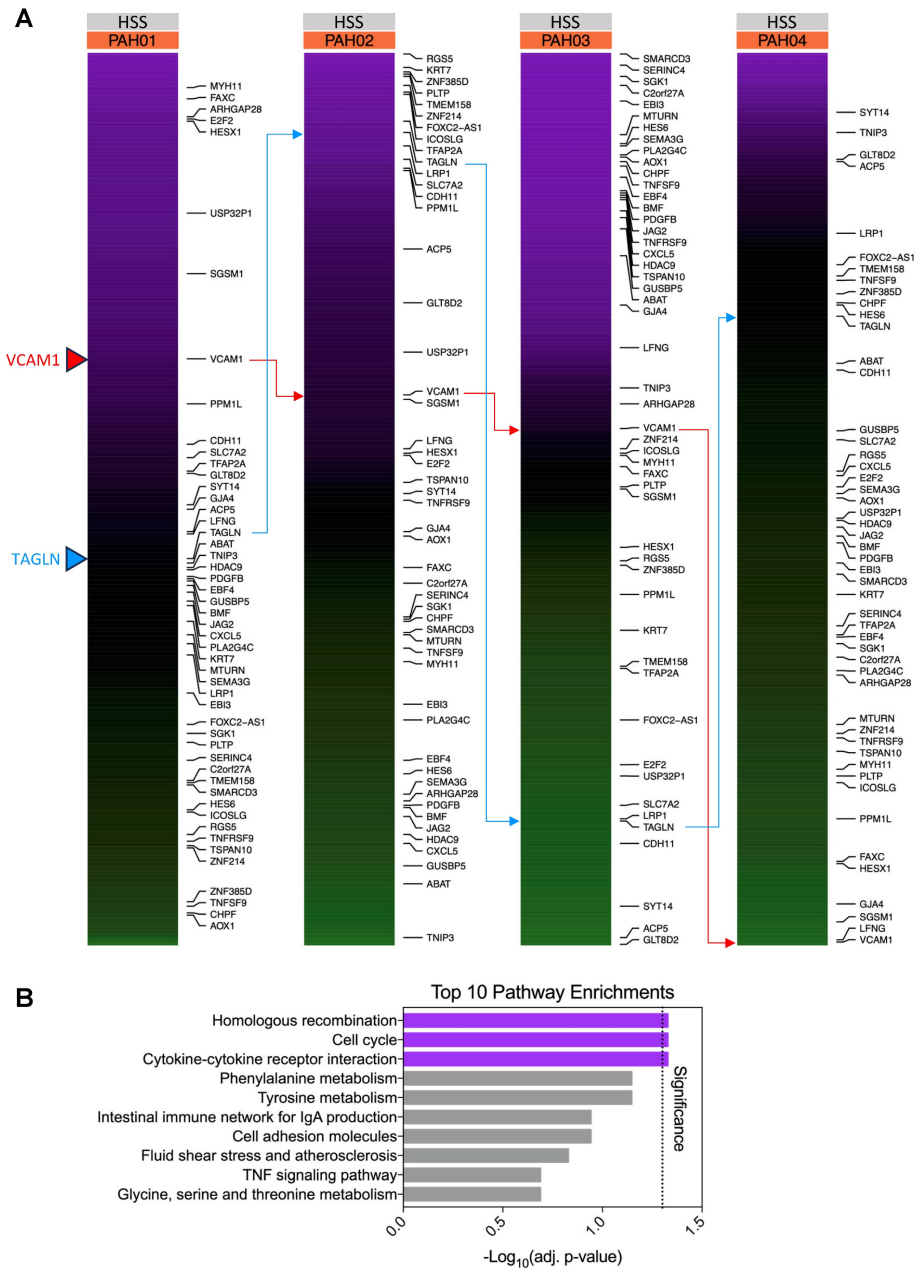
** Gene variability analysis reveals the main drivers of heterogeneity in PAH hPMECs unveiled by increasing shear stress.** (A) Comparing the “top variable genes” in terms of log_2_CPM-based HSS z-scores, the expression of the genes between patients is seen to be highly variable as expected. For example, *VCAM1* is comparatively highly expressed in PAH01, relatively near the mean in PAH02 and PAH03, and comparatively lowly expressed in PAH04. Meanwhile *TAGLN* is comparatively highly expressed in PAH02, relatively near the mean in PAH01 and PAH04, and comparatively lowly expressed in PAH03. The top 50 ∆∆CV genes are shown on the right, further demonstrating the shifting distribution of these genes between patients. Figure generated using the *ComplexHeatmap 2.14.0* R package. (B) Over-representation pathway enrichment analysis of the top 398 variable genes in PAH due to HSS identified few significantly enriched pathways: homologous recombination (p=0.047, 6 genes), cell cycle (p=0.047, 11 genes), and cytokine-cytokine receptor interaction (p=0.047, 12 genes).

Subsequently, we calculated z-scores for each of the “top variable genes” HSS log_2_CPM values across the four PAH patients, focusing on the HSS condition due to its greatest observed inter-patient heterogeneity (Figure 4A, Table S4). This z-score standardization emphasized considerable differences in the relative rankings of these genes between patients, further demonstrating the role of these genes in driving inter-patient heterogeneity.

This inter-patient heterogeneity is further emphasized when we perform an over-representation analysis on these “top variable” genes, where, as expected, minimal significant pathway enrichments are identified (homologous recombination (p=0.047, 6 genes), cell cycle (p=0.047, 11 genes), and cytokine-cytokine receptor interactions (p=0.047, 12 genes), Figure 4B, Table S5). The substantial differences in relative gene expressions between these patients suggest that individual gene regulation patterns need to be considered to capture patient-specific signatures.

### Unsupervised clustering on PAH top variable genes in response to shear stress reveals patient-specific disease signatures

**Figure 5:**
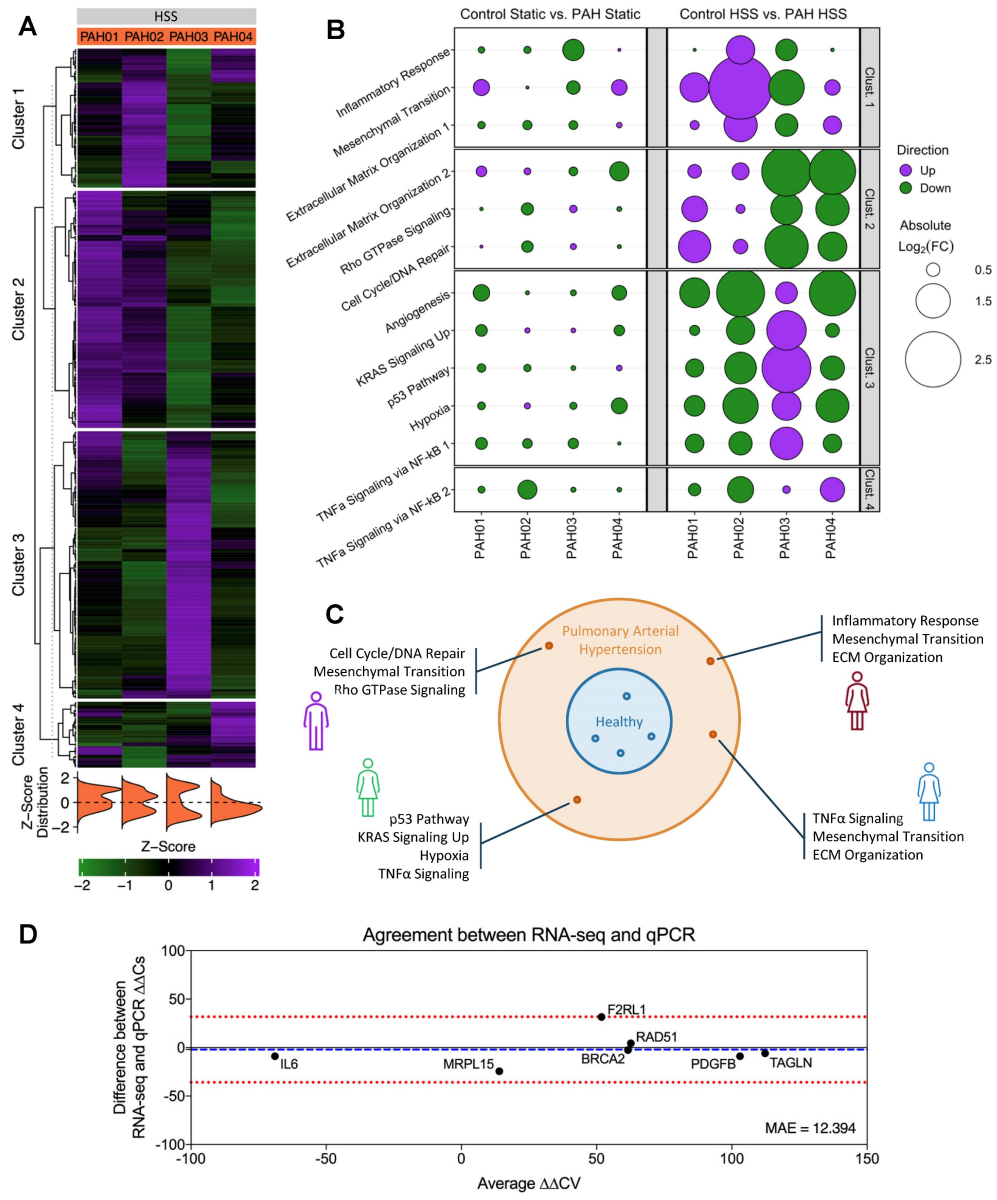
** Unsupervised clustering on PAH “top variable genes” in response to shear stress reveals patient-specific disease signatures.** (A) Unsupervised PAM clustering on PAH log_2_CPM-based z-scores for “top variable genes”. Clear patient-specific signatures were observed, with each cluster correlating strongly to one individual patient. Figure generated using the *ComplexHeatmap 2.14.0* R package. (B) Pathway enrichment analysis for each cluster gene set identified cluster-specific significantly enriched pathways. Bubble size indicates the magnitude of average log_2_FC of the “top variable genes” in each pathway, quantified as individual patient *vs.* grouped control. PAH hPMECs displayed patient-specific and exacerbated PAH signaling under HSS not seen under Static conditions or in grouped analyses. Figure generated using the *ggplot2 3.5.1* R package. (C) Individual PAH patient pathological signatures were able to be extracted from transcriptional heterogeneity of hPMECs in response to HSS, stratifying patients on a molecular basis. (D) Key cluster-defining genes were validated via qPCR using hPMECs from the same patients with the same stimuli, showing a minimal MAE (12.394) between qPCR and RNA-seq ∆∆CV. Bland-Altman analysis demonstrated a lack of systemic biases or patterns in the differences between methods, pointing to the identified variability as biological heterogeneity (Bias: -2.09; 95% Limits of Agreement: [-35.9, 31.73]).

In order to separate individual patients and unveil their specific differential responses to HSS, unsupervised PAM clustering was applied to the z-scores of PAH HSS “top variable genes” calculated previously in Table S4 (Figure S9, Table S6). The resulting clusters revealed distinct upregulated gene profiles for each patient, with minimal similarity between patients (Figure 5A, Table S7).

Using these clusters as gene sets, significant pathway enrichments were identified while ensuring minimal gene overlap between pathways (Figure S10). Cluster 1 showed Inflammatory Response (p=0.01648), Epithelial Mesenchymal Transition (p=0.00002173), and Extracellular Matrix Organization R-HSA-1474244 (p=0.04558) significantly enriched. Cluster 2, Extracellular Matrix Organization R-HSA-1474244 (p=0.03022), Rho GTPase Signaling [incl. Rho GTPase Effectors R-HSA-195258 (p=3.72E-04), Rho GTPases Activate Formins R-HSA-5663220 (p=8.09E-04), and Signaling by Rho GTPases R-HSA-194315 (p=0.032517)], and 70 significantly enriched pathways related to cell cycle and DNA repair [*e.g.*, Cell Cycle (p=1.56E-05), Homologous Recombination (p=4.42E-05), G2-M Checkpoint (p=1.75E-19), and E2F Targets (p=4.08E-09)]. Cluster 3 Angiogenesis (p=0.02804), KRAS Signaling Up (p=0.01394), p53 Pathway (p=0.008256), Hypoxia (p=0.008256), and TNFα Signaling via NF-κB (p=3.134E-06). Cluster 4 only showed enrichment in TNFα Signaling via NF-κB (p=0.02247).

Utilizing these identified pathways to assess the magnitude of individual patient pathway dysregulation, we then calculated the average log_2_FC for genes contributing to each pathway's significant enrichment, comparing individual patients *vs.* grouped controls (Figure 5B). Matching closely to the relative differences observed in Figure 5A, similar patterns emerge for individual patients against controls. From this, distinct defining upregulations were noted for individual patients: PAH01 upregulated Cluster 2 pathways, PAH02 upregulated Cluster 1 pathways, PAH03 upregulated Cluster 3 pathways, and PAH04 upregulated Cluster 4 pathways.

An alternative approach to identifying patient-specific signatures was conducted using the unclustered “top variable genes” in a gene set enrichment analysis (GSEA) 25 ranked by descending z-scores for each patient (Figure S11). While this approach identified many of the same patient-specific signatures and provides useful insights into pathway-level dysregulation, its focus on isolated individual patient ranked lists limits the ability to detect potential sub-groups of patients with shared molecular profiles, thus demonstrating how clustering promotes a more nuanced understanding of the disease's heterogeneity.

In summary, through this variability analysis, we assessed healthy biological variability and identified genes from the PAH cohort that deviated from this baseline. Unsupervised clustering of these genes then facilitated the identification of patient-specific pathways (Figure 5C).

To validate our measurements, we performed qPCR on matched hPMEC samples under identical shear conditions. Genes were selected from the identified clusters, with a focus on those representing key pathways that could, independently or in combination, uniquely identify patients, while also exhibiting substantial divergence in the log_2_FC across the four patients (*BRCA2*, *F2RL1*, *RAD51*, *PDGFB*, and *TAGLN*). Additionally, we included a negative non-shear-responsive control (*MRPL15*) and an oppositely directed ∆∆CV gene (*IL6*). The ∆∆CV (Equation 1, Equation 2) of these genes were calculated and compared to RNA-seq data using Mean Absolute Error (MAE) and a Bland-Altman plot (Figure 5D). The MAE of 12.394 indicates a minor average ∆∆CV discrepancy between the two methods, and the Bland-Altman analysis confirmed a high level of agreement across a wide range of ∆∆CV values, supporting the reliability of our transcriptomics results in capturing true biological heterogeneity (Bias: -2.09; 95% Limits of Agreement: [-35.9, 31.73]). Furthermore, control HSS *vs.* PAH HSS fold changes showed low discrepancy (MAE) and strong linear correlation (Pearson r) between RNA-seq and qPCR, reinforcing the identified magnitudes of pathway dysregulations (Figure S12).

### Confirmation of hPMEC gene expression patterns in PBMCs supports translatability of findings to clinically accessible material

Recognizing the systemic impact of altered hemodynamics in PAH, we extended our analysis to PBMCs. As PBMCs are exposed to the hemodynamically altered circulation of the PAH lung, we hypothesized that these circulating cells may also exhibit comparable transcriptional changes. By investigating PBMCs, we aimed not only to validate our findings in a larger cohort but also to provide a clinically accessible diagnostic approach to identifying patient-specific disease signatures.

**Figure 6:**
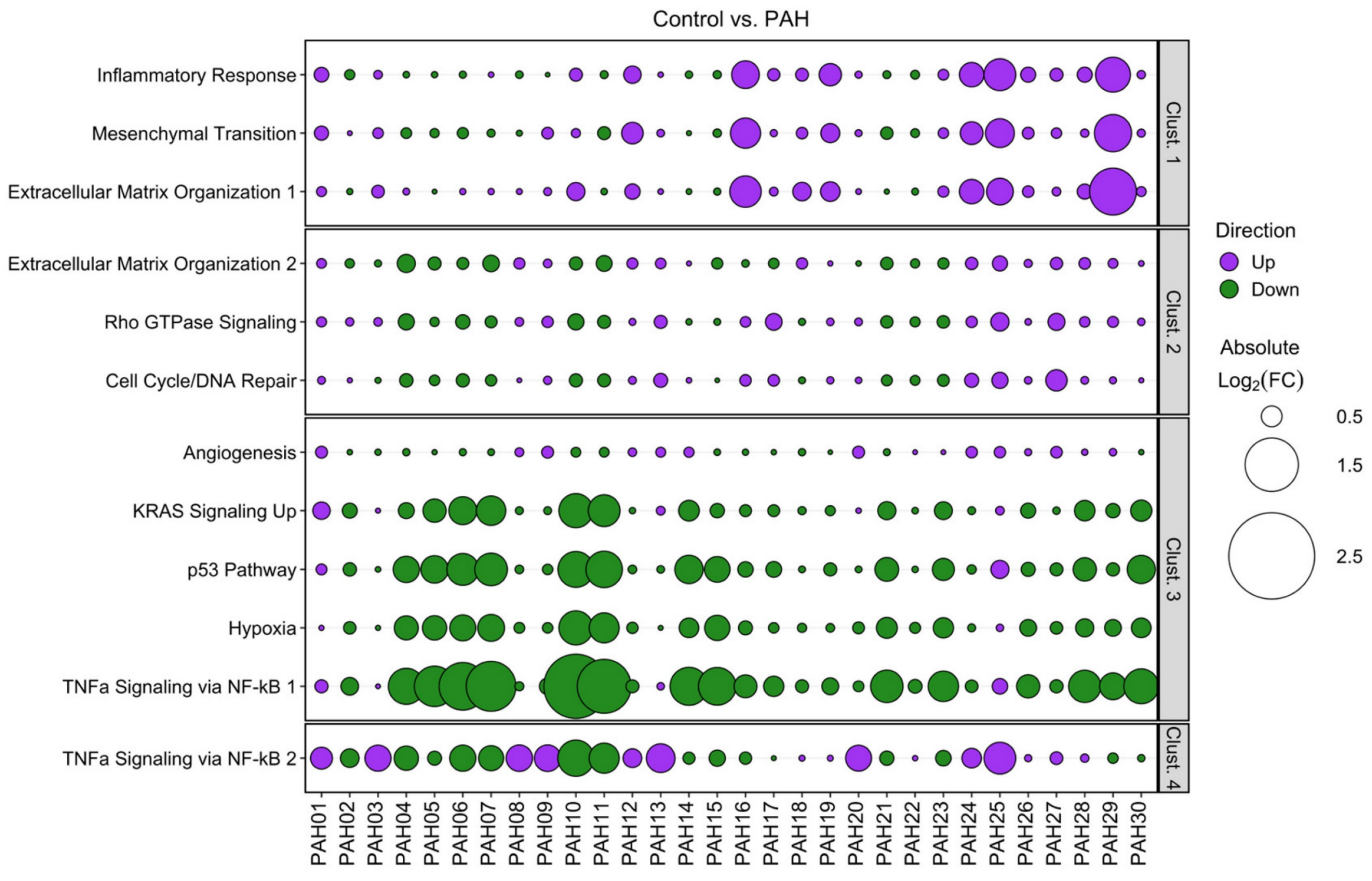
** Validation of hPMEC gene expression patterns in PBMCs supports translatability of findings to clinically accessible material for patient sub-stratification.** Using a publicly-available PAH PBMC microarray dataset (GEO accession: GSE33463) 21, patient-specific fold changes of the previously-identified cluster pathways, utilizing the same “top variable genes” from our earlier analysis, were calculated. Intra-cluster expression trends remained consistent, while inter-patient differences were still evident and could be used to identify individual patients or patient sub-groups. Clusters 1, 3, and 4 demonstrated the greatest capacity to retain PAH patient stratification in PBMCs. Figure generated using the *ggplot2 3.5.1* R package.

Analysis of the published PBMC microarray dataset (GEO accession: GSE33463 21) focused on the same clusters and pathways identified in our PAH patient hPMECs. We found that the same expression trends and inter-patient cluster differences were consistently reproduced in the PAH PBMCs compared to controls, although the magnitude of pathway dysregulations and divergence between patients was less pronounced (Figure 6). Nevertheless, the capacity to discern individual patients/patient sub-groups was retained, especially through clusters 1, 3, and 4. This consistency in patient-specific variations confirms that the shear stress-induced gene expression patterns in PAH hPMECs are effectively reflected in PAH PBMCs, emphasizing the applicability of our identified clusters and the capacity of our methodology to uncover individual patient and patient sub-group pathogenotypes.

## Discussion

This study advances our understanding of the diverse molecular mechanisms underlying PAH by focusing on two key contributions. First, we integrated bulk transcriptomic sequencing of PAH and control hPMECs with exposure to shear stress *in vitro*, as a novel contribution to the field. Second, we developed a transcriptomic analytical strategy to uncover patient-specific mechanisms in PAH, providing deeper insights into the heterogeneity of this disease. Endothelial dysfunction is a key factor in PAH pathogenesis, with evidence demonstrating that endothelial cells from PAH patients exhibit abnormal responses to hemodynamic forces, disrupting vascular homeostasis 26-29. We aimed to investigate this dysfunction transcriptionally to address the diagnostic and therapeutic challenges in PAH, where large-scale clinical trials have struggled to identify consistent molecular denominators across patients 3-6. Our findings show that considering patients as a homogeneous group obscures key molecular insights, as grouped analyses fail to capture the patient-specific complexity of PAH. This aligns with studies recognizing the molecular and phenotypic diversity in PAH 30, 31, and highlights the variability in disease progression and treatment responses reported in clinical registries 32-35.

A central insight from our study is that HSS, modeling the pathological mechanical forces in PAH, reveals patient-specific molecular differences in hPMECs. Shear stress is known to modulate endothelial function and gene regulation, particularly in processes such as inflammation, angiogenesis, and extracellular matrix remodeling 11, 36. For instance, laminar shear stress promotes an anti-inflammatory endothelial phenotype, while disturbed flow leads to endothelial dysfunction and inflammation as seen in atherosclerosis 37. Our findings extend this by showing that individual patient hPMEC responses to supra-physiological shear stress vary significantly in PAH, contributing to the patient-specific molecular heterogeneity observed in the disease.

Through our analysis, we identified patient-specific gene expression patterns, and patient-differentiating genes such as *RAD51*, *BRCA2*, *TAGLN*, *PDGFB*, and *F2RL1*. These genes were part of larger identified gene subsets affecting key PAH-associated pathways such as cell cycle and DNA repair, mesenchymal transition, inflammatory response, extracellular matrix organization, and TNFα signaling. Notably, each patient exhibited distinct and often divergent combinations of these pathways, reinforcing the need for personalized approaches and individualized patient stratification. Previous research has highlighted the relevance of these pathways in PAH, particularly their role in endothelial dysfunction, vascular remodeling, and inflammation 28, 38-40.

*BRCA2* and *RAD51*, genes part of the larger dysregulated pathways of cell cycle regulation and DNA repair, emerged as key patient-defining markers. *RAD51* 41, and more broadly, DNA repair pathways have been linked to PAH, contributing to dysregulated cellular senescence, proliferation, and apoptosis resistance 42, 43. Elevated *TAGLN*, an early marker of mesenchymal transition, may indicate a sub-group of patients prone to exacerbated vascular remodeling and stiffness 44. *PDGFB*, involved in vascular smooth muscle cell proliferation and ECM remodeling, may identify patients predisposed to aggressive medial hypertrophy, aligning with evidence that excessive *PDGF* signaling drives pathological vascular remodeling in PAH 45. Its connection to hypoxia, a known driver of PAH, further highlights the value of stratifying patients based on *PDGF* signaling, which has already been targeted therapeutically in PAH with imatinib 46. Less explored in PAH, *F2RL1* (*PAR2*) is linked to vascular smooth muscle relaxation 47, immune-mediated damage 48, and TNFα signaling 49, suggesting patients with altered *F2RL1* expression may experience heightened immune responses and dysregulated vascular tone. Rivaroxaban has also previously been shown to downregulate *F2RL1* and associated pathways (ERK, JNK, NF-κB), thereby attenuating right ventricular remodeling in a PAH Sugen-Hypoxia rat model, indicating roles of *F2RL1* in vascular remodeling by various mechanisms, fibrosis, and endothelial dysfunction in PAH 50, 51.

The validation of our findings in PBMCs enhances their translational relevance. PBMCs are recognized as non-invasive biomarkers in cardiovascular diseases, including PAH, where they can reflect systemic inflammation and endothelial dysfunction 52, 53. The identification of similar gene expression patterns in PBMCs, particularly in genes involved in pathways such as KRAS signaling, p53 pathway, TNFα signaling, and hypoxia response, underscores the utility of our approach for PBMC-based molecular diagnostics in PAH. Prior studies have also demonstrated the ability to identify PAH patients based on PBMC gene expression profiles with high certainty, supporting their potential for patient stratification based on molecular risk profiles and gene patterns 21, 22, 54.

Our findings hold important implications for improving PAH patient selection for treatment by identifying molecular profiles that classify patients based on activity of transcriptomic patterns. The molecular heterogeneity we observed may help explain the variability in treatment responses seen in PAH, where therapies like endothelin receptor antagonists and phosphodiesterase type 5 inhibitors have produced inconsistent outcomes 55, 56. Understanding patient-specific molecular signatures could therefore assist in guiding therapeutic decisions and improve outcomes. This personalized approach aligns with advances in oncology, where molecular stratification of patients has increasingly been emphasized for clinical trial design and drug development 57.

While our study offers important insights into PAH's molecular heterogeneity, several questions remain to be further explored. A key limitation is that our approach may not capture gene patterns broadly shared across the cohort, focusing instead on signatures that distinguish individual patients from the norm. Incorporating more clinically well-defined and diverse samples, including healthy controls, could refine PAH signatures and better capture underlying pathological patterns. Specifically, due to the small sample size, we were unable to consider sex differences in our analysis. However, as the female sex has historically been considered a risk factor, our sample set was selected to match the clinical situation of ~75% female patients 32. Additionally, while PBMCs were used to validate gene patterns, they were not derived from the same patients as the hPMECs. This makes it unclear to what extent PBMC gene variability reflects endothelial variability in the same individuals. Therefore, future prospective studies correlating PBMC and hPMEC data from the same patients could provide deeper insight into how circulating cells mirror tissue resident endothelial gene expression. Additionally, the published data set does not include information on clinical phenotype, treatment regimen, or drug efficacy, making correlations with the patient signatures not possible.

Moreover, our study does not investigate the long-term evolution of the shear-induced transcriptomic responses observed in PAH. Longitudinal studies that track gene expression changes in both endothelial cells and PBMCs would therefore offer important insights into how patient-specific signatures could influence or forecast disease progression and treatment outcomes. Further exploring the relationship between shear stress responses and clinical metrics, such as hemodynamic measurements and treatment regimens, may also provide a more nuanced understanding of PAH prognosis. Therefore, integrating findings from our analysis pipeline with clinical metrics could enhance predictions of patient-specific disease progression and response to therapy, potentially improving patient management strategies and long-term outcomes.

## Conclusions

Our study characterizes the transcriptomic landscape of hPMECs in PAH under varying shear stress conditions, leveraging shear stress as both a hallmark of PAH pathophysiology and a key regulator of endothelial function and vascular homeostasis 11. Our analysis captured the complexity and variability of hPMEC responses to supra-physiological shear stress, revealing distinct molecular profiles for individual patients. These patient signatures were translatable to clinically accessible PBMCs, further enabling patient stratification based on the identified heterogeneity. By exploring molecular variability in PAH, our study advances patient molecular sub-classification and highlights the importance of personalized molecular insights, establishing a foundation for improved management and individualized therapeutic treatments in this clinically well-defined yet molecularly heterogeneous disease.

## Supplementary Material

Supplementary figures.

Supplementary tables.

Supplementary fragments per gene.

## Data availability

RNA-sequencing data is available as “fragments_per_gene.xlsx” in the Supplementary Material.

## Figures and Tables

**Table 1:** Control donor and PAH patient characteristics.

Control group hPMECs										
*ID*	*Assays*	*Diagnosis*	*FVC*	*FEV1*	*Dyspnea*	*Sex*	*Age*	*Ethnicity*	*Source*	*Echo/CT*
CTR01	RNAseq, qPCR	NSCLC, squamous cell carcinoma	2.75 (100%)	1.17 (50%)	No	F	61	Caucasian	Lob	No dilation of RV, RA, or LV
CTR02	RNAseq, qPCR	NSCLC, squamous cell carcinoma	3.11 (100%)	2.31 (98%)	No	M	79	Caucasian	Lob	No dilation of RV, RA, or LV
CTR03	RNAseq, qPCR	NSCLC, adeno-carcinoma	-	-	No	F	55	Caucasian	Lob	No dilation of RV, RA, or LV
CTR04	RNAseq, qPCR	Tumoral obstruction	-	-	Yes	M	42	Caucasian	Lob	Enlarged RV, small LV, enlarged RA
PAH patient hPMECs										
*ID*	*Assays*	*Diagnosis*	*mPAP*	*PVR*	*CI*	*Sex*	*Age*	*Ethnicity*	*Source*	*Treatment*
PAH01	RNAseq, qPCR	iPAH	102	1375	3.4	M	21	Caucasian	Ltx	PDE5-I, ERA, PGI2
PAH02	RNAseq, qPCR	hPAH (*BMPR2*)	68	-	1.6	F	40	Caucasian	Ltx	PDE5-I, ERA, PGI2
PAH03	RNAseq, qPCR	iPAH	54	-	2.1	F	54	Caucasian	Obd	PDE5-I, ERA, PGI2
PAH04	RNAseq, qPCR	iPAH	43	620	2.1	F	42	Caucasian	Ltx	PDE5-I, PGI2

hPMEC = human pulmonary microvascular endothelial cells; NSCLC = non-small-cell-lung carcinoma; FVC = forced vital capacity (L); FEV1 = first second of forced expiration (L); RV = right ventricle; RA = right atrium; LV = left ventricle; iPAH = idiopathic pulmonary arterial hypertension; hPAH = hereditary pulmonary arterial hypertension; mPAP = mean pulmonary artery pressure (mmHg); PVR = pulmonary vascular resistance (WU); CI = cardiac index (l/min/m²); PDE5-I = phosphodiesterase type 5 inhibitor; PGI2 = prostacyclin; ERA = endothelin receptor antagonist; Lob = lobectomy; Obd = autopsy; Ltx = lung transplantation.

## Abbreviations

CCA: canonical correlation analysis
CPM: counts per million
CV: coefficient of variation
DEG: differentially-expressed gene
FC: fold change
GEO: Gene Expression Omnibus
GSEA: gene set enrichment analysis
HSS: high shear stress
LSS: low shear stress
MAE: mean absolute error
PAH: pulmonary arterial hypertension
PAM: partitioning around medoids
PBMC: peripheral blood mononuclear cell
PCA: principal component analysis
hPMEC: human pulmonary microvascular endothelial cell
qPCR: quantitative real-time polymerase chain reaction
