# Supplementary material for: Shear stress unveils patient-specific transcriptional signatures in PAH: Towards personalized molecular diagnostics
Source: Theranostics. 2025 Jan 2;15(5):1589–605. doi: 10.7150/thno.105729 (PMC11780538; doi:10.7150/thno.105729)
Supplement: Supplementary file 1 — Supplementary figures. [file thnov15p1589s1.pdf]

Supplementary Figures:

Shear stress unveils patient-specific transcriptional signatures in PAH: Towards personalized molecular diagnostics

Corey Wittig, Jakob M König, Xiaoke Pan, Jurjan Aman, Harm-Jan Bogaard, Paul B Yu, Wolfgang M Kuebler, Katharina Baum, and Robert Szulcek

This file includes:

- Figures S1-S12

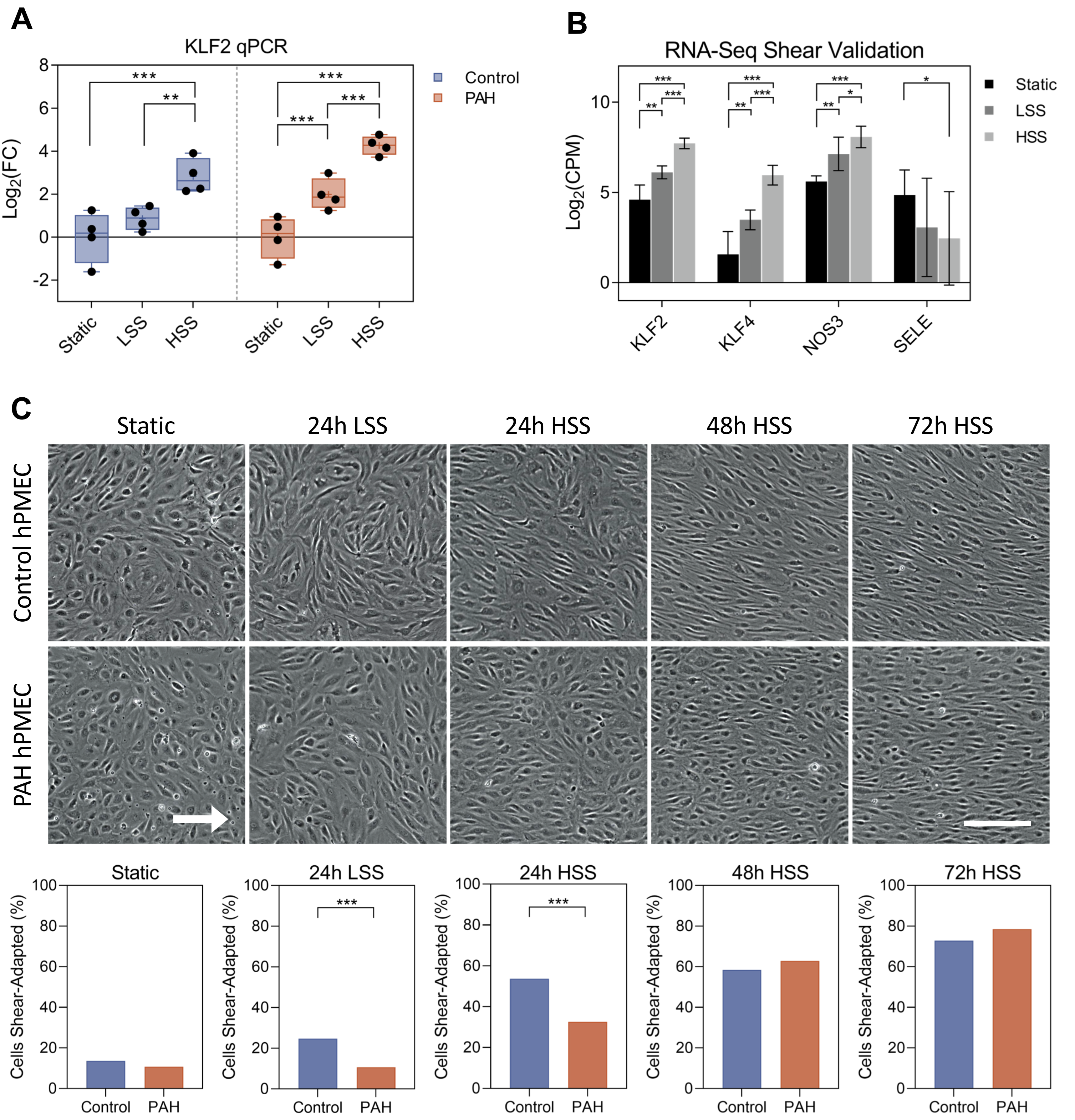

Figure S1: Validation of shear sensing of hPMECs. (A) Using qPCR, *KLF2* was seen to be significantly upregulated from Static to HSS for both control and PAH hPMECs (control: $p < 0.001$, $\log_2FC=2.83$; PAH: $p < 0.001$, $\log_2FC=4.26$) and from LSS to HSS (control: $p=0.002$, $\log_2FC=1.96$; PAH: $p < 0.001$, $\log_2FC=2.27$), demonstrating hPMECs properly sensed the applied fluid shear stress. *KLF2* was also significantly upregulated from Static to LSS in PAH hPMECs (control: $p=0.08$, $\log_2FC=0.87$; PAH: $p < 0.001$, $\log_2FC=1.99$). Boxes = 25th-75th percentiles, whiskers = min-max, mean = "+", median = horizontal line. **(B)** In the RNA-seq dataset, numerous known shear-sensitive genes were seen to significantly respond to shear stress in a shear magnitude-dependent manner. Data displayed as mean ± SD (n=8/group). **(C)** Visualization and quantification of hPMEC morphology demonstrates responsivity of both control and PAH hPMEC to the applied shear stress via elongation and alignment with the direction of fluid flow after 48-72h HSS. PAH hPMEC were delayed in their morphological adaptation to HSS compared to controls, in line with our previous work [ref. 15]. 500 cells were measured per condition, and p-values were calculated using Fisher's Exact Test. Scale bar = 200 µm. Shear-adapted = aspect ratio (l/w) ≥ 3.0 and <30˚ orientation to direction of fluid flow.

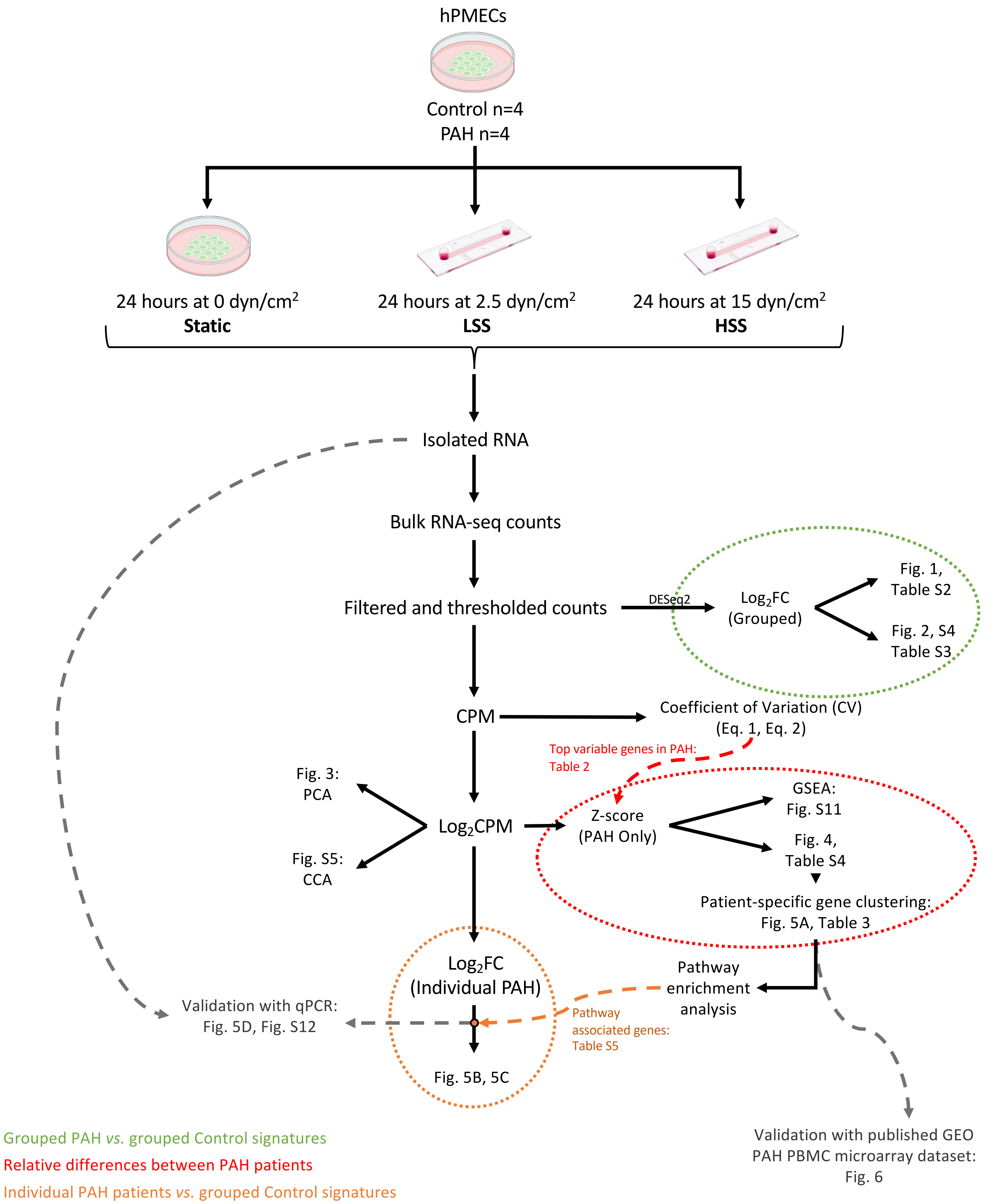

Figure S2: Data curation and analysis pipeline. Steps taken to generate and process the dataset for each form of analysis. hPMEC = human pulmonary microvascular endothelial cells, PAH = pulmonary arterial hypertension, LSS = low shear stress, HSS = high shear stress, CPM = counts per million, FC = fold change, PCA = principal component analysis, CCA = canonical correlation analysis, GSEA = gene set enrichment analysis, GEO = gene expression omnibus, PBMC = peripheral blood mononuclear cells.

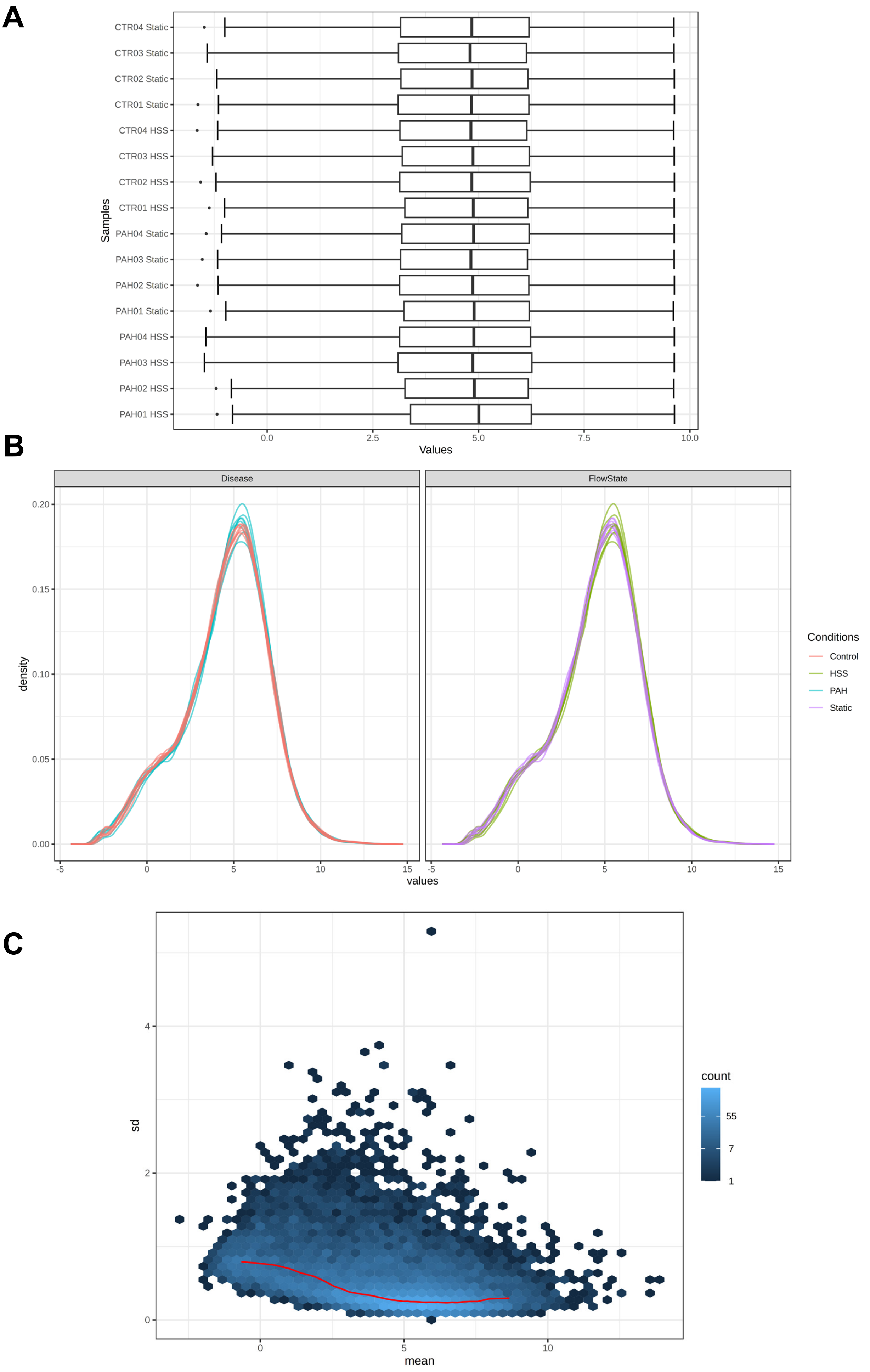

Figure S3: Dataset normalization and quality control. (A) Dataset log_2CPM normalization produced comparable samples. **(B)** The distribution of normalized values in control, PAH, static, and HSS samples demonstrated a similar profile with generally normal distributions. **(C)** The normalized log_2CPM expression values have minimal correlation between the gene expression mean and standard deviation. Figures generated using ExpressAnalyst.

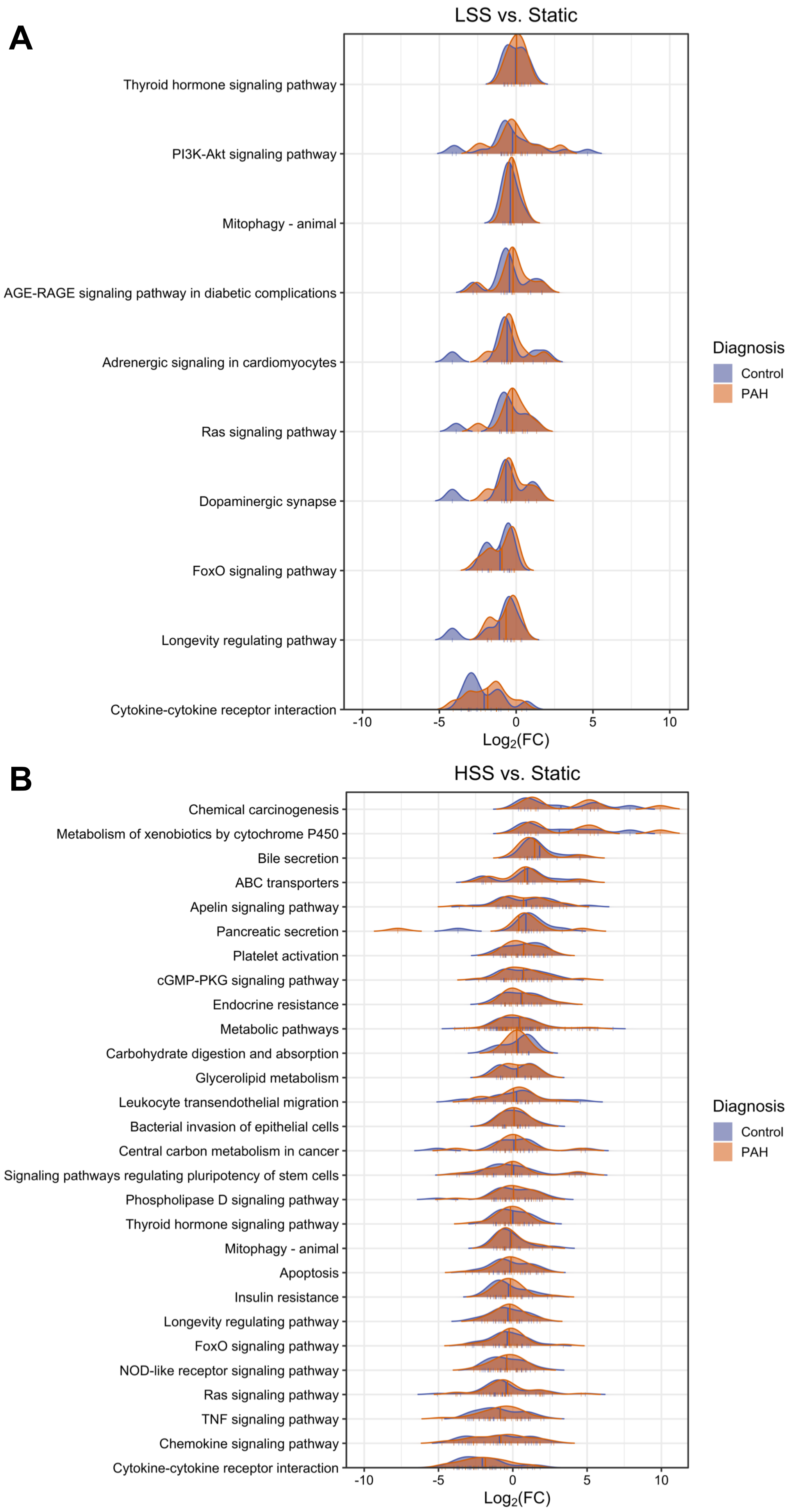

Figure S4: Overlapping flow response patterns between control and PAH for pathways significantly changed only in controls demonstrate overall conserved flow responses in PAH despite lacking significance. Density plots for each significantly changed pathway in control flow responses, but not significantly changed in PAH flow responses. The curves represent the expression change profile in terms of log_2FC of each individual DEG in the pathway (marked as ticks on pathway x-axes). Mean pathway log_2FC are marked by a vertical line. **(A)** LSS *vs*. Static **(B)** HSS *vs.* Static. Figure generated using the *ggridges 0.5.6* R package.

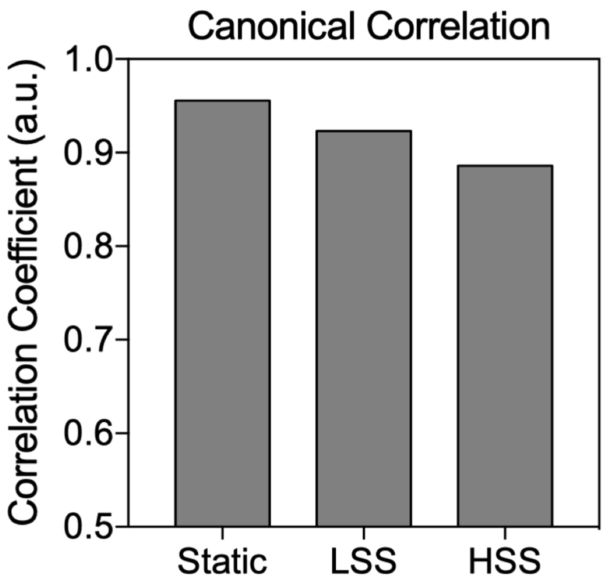

Figure S5: Canonical correlation analysis demonstrates PAH and control hPMECs become transcriptionally less similar with increasing shear stress. Three-component canonical correlation analysis was performed on the full filtered transcriptome of control *vs.* PAH at each flow condition. The decreasing correlation coefficient with increasing shear stress indicates a weaker alignment of gene expression patterns, indicating lower similarity.

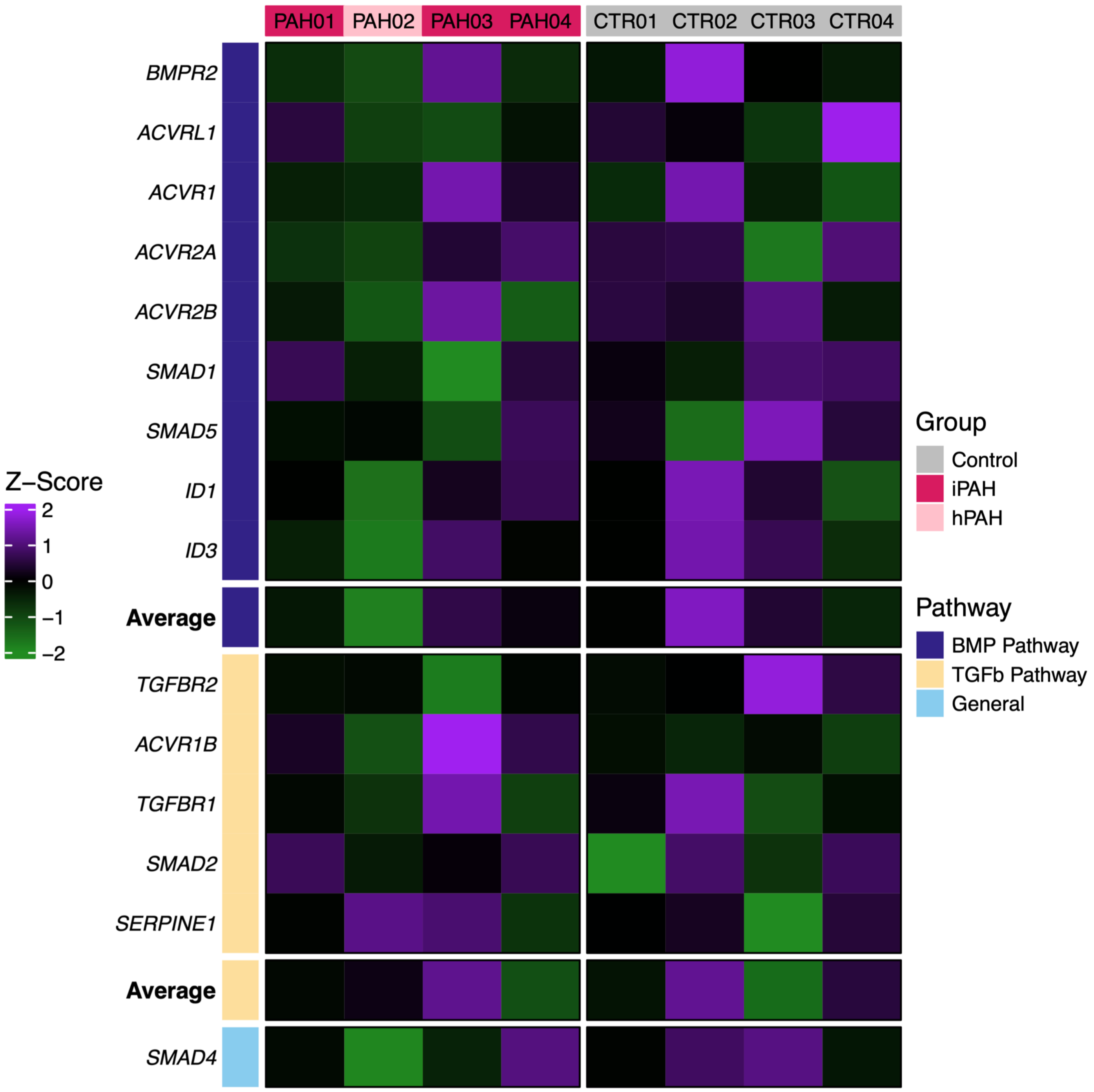

Figure S6: HSS exacerbates PAH02's known BMP signaling dysregulation. Fold changes from static to HSS were calculated on an individual sample basis for genes involved in BMP or TGFb signaling, then z-scored across all samples. HSS amplified imbalanced BMP-TGFb signaling in PAH02 (which harbors a *BMPR2* loss-of-function mutation), with downregulated BMP signaling and unchanged TGFb signaling in response to HSS. This effect was not seen in the other patients or controls, demonstrating that HSS exacerbates patient-specific underlying PAH transcriptional patterns. Figure generated using the *ComplexHeatmap 2.14.0* R package.

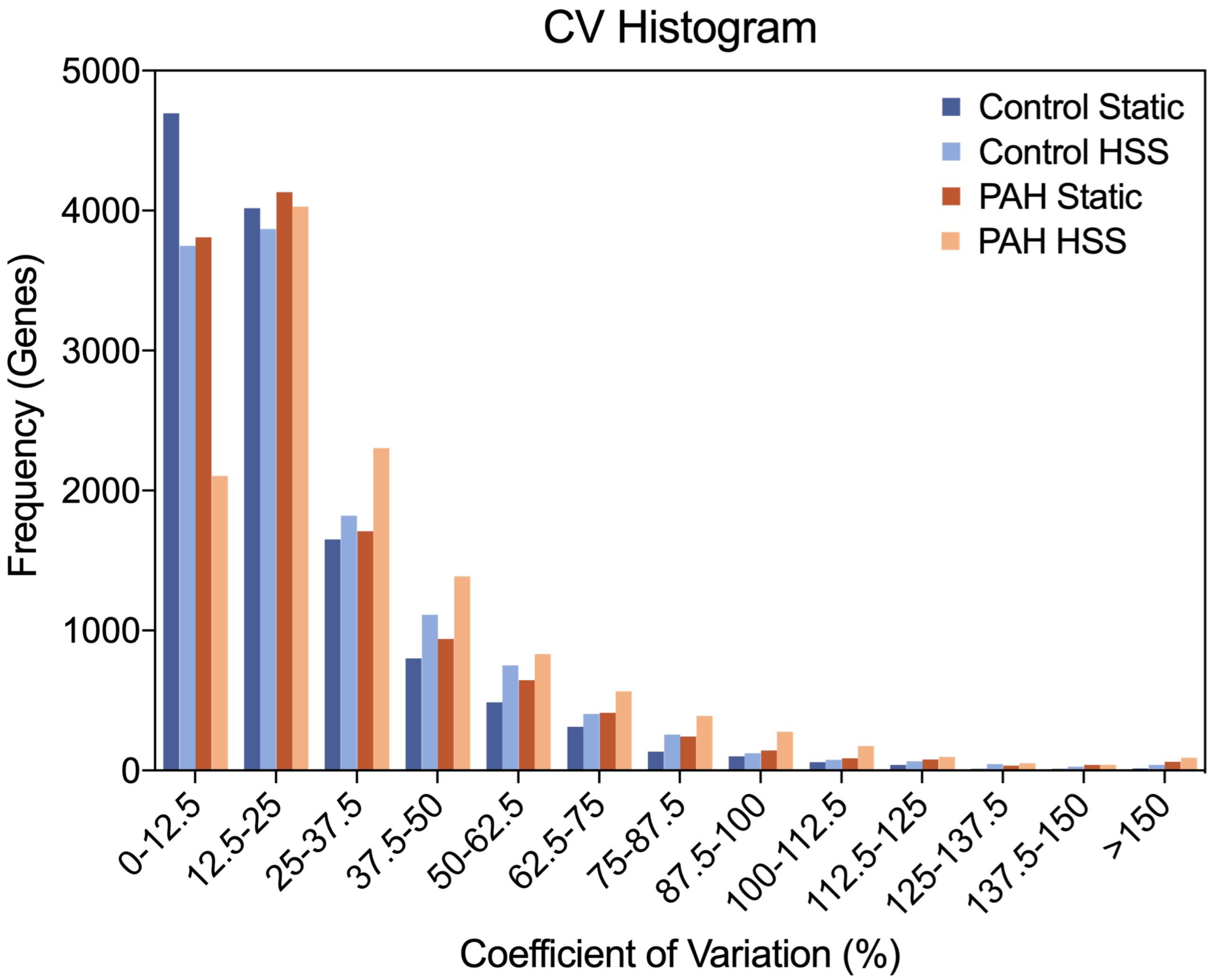

Figure S7: Validation of coefficient of variation distribution skewing due to HSS in PAH hPMECs. The frequency of genes with a high coefficient of variation is skewed higher in the PAH HSS condition compared to other conditions, consistent with the finding that HSS increases variability in PAH in a pathological manner.

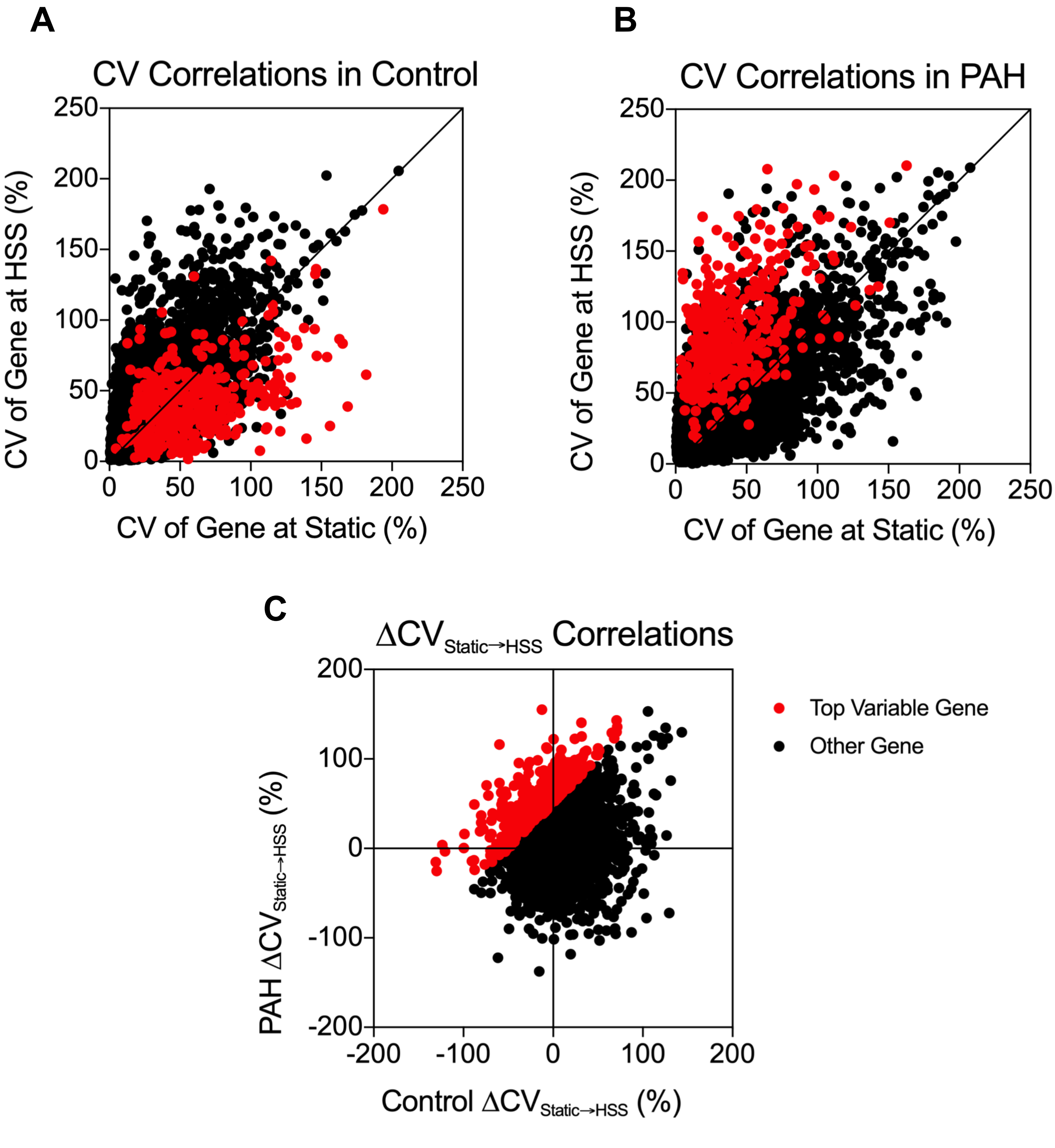

Figure S8: Intermediate processing undertaken to determine "top variable genes". (A) Control CV (coefficient of variation), **(B)** PAH CV, and **(C)** ΔCV (CV_{HSS} - CV_{Static}) correlations for intermediate calculations undertaken to determine "top variable genes". "Top variable genes" are shown in red.

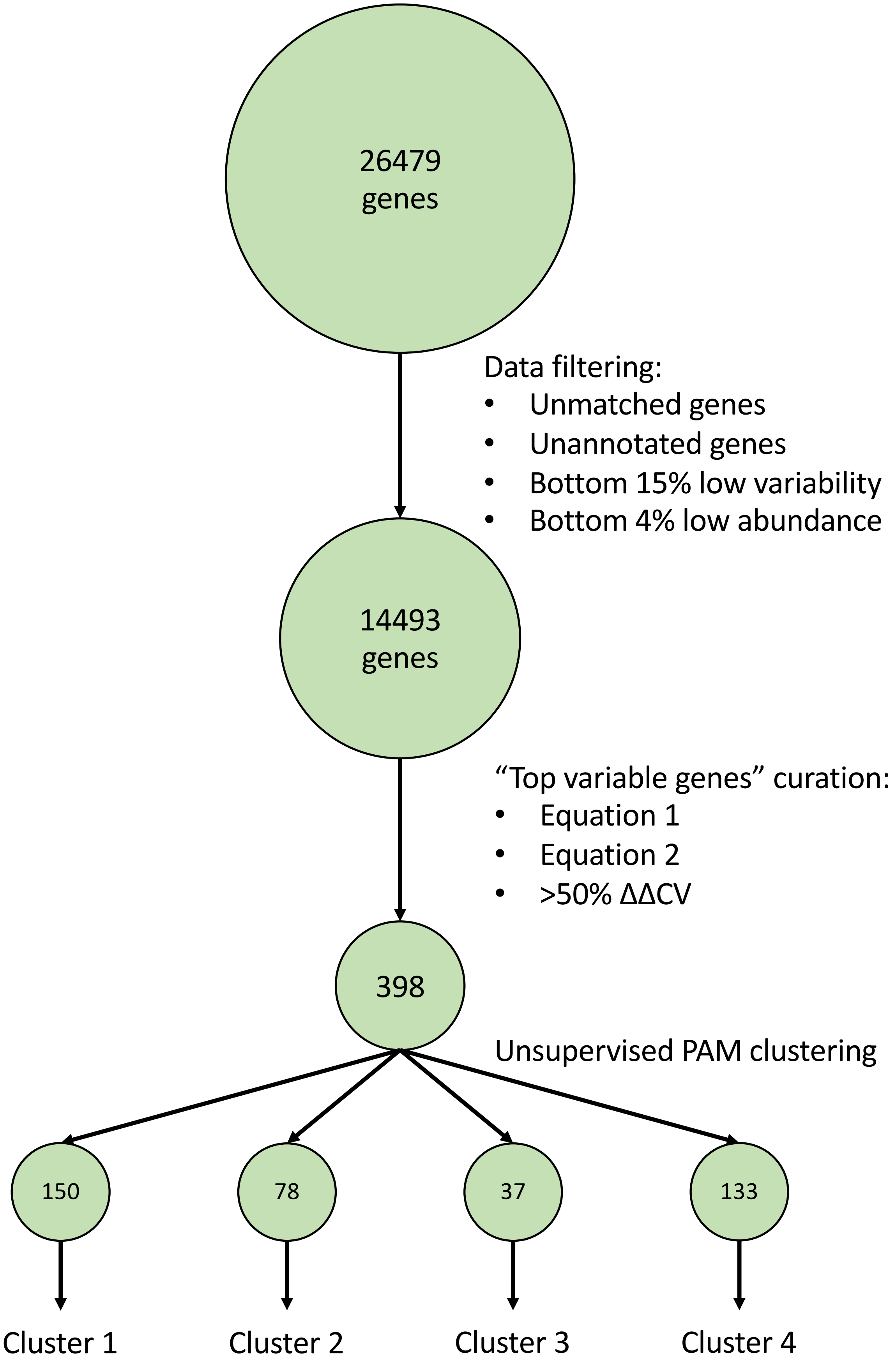

Figure S9: Dataset refinement to specific gene clusters of individual dysregulations. The dataset was reduced from 26,479 genes to 14,493 genes through data filtering, then further to the 398 “top variable genes” based on >50% ΔΔCV score. Clustering on these 398 “top variable genes” produced four clusters of genes, ranging from 37 to 150 genes. Over-representation analysis on these cluster gene sets produced individual signatures of dysregulation that could be correlated to individual patients.

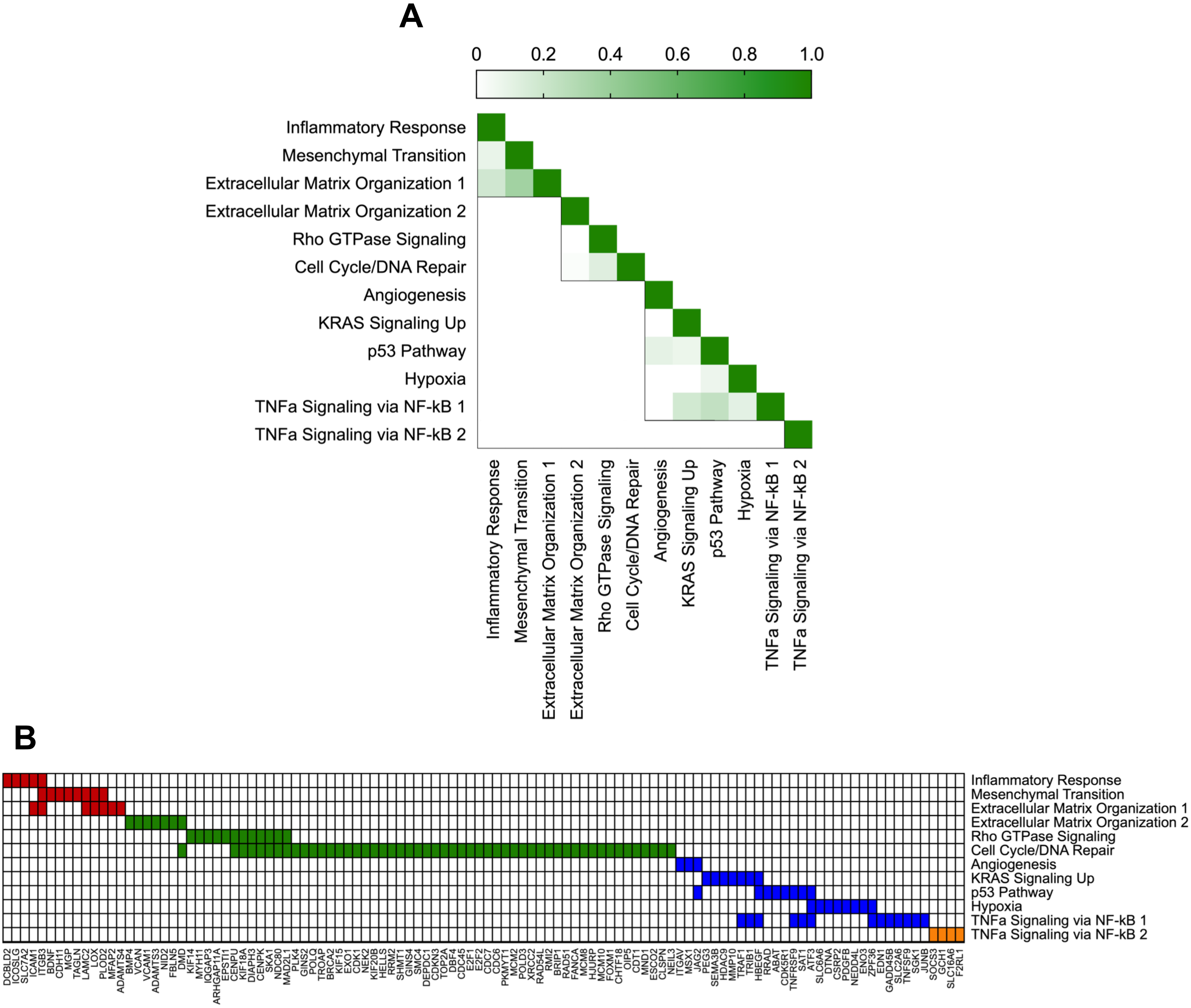

Figure S10: Overlap of genes in significantly enriched pathways from "top variable genes" clustering. (A) Set-in-set gene overlap was determined for the significantly enriched pathways in each PAM cluster, using only the "top variable genes". Score is calculated as the ratio of intersection of genes in the two pathways and union of genes in the two pathways. A score of 1 indicates complete overlap of genes in the two pathways, while a score of 0 indicates no overlapping genes. **(B)** Genes in each significantly enriched pathway derived from PAM clustering. Red = cluster 1 pathways, green = cluster 2 pathways, blue = cluster 3 pathways, orange = cluster 4 pathway.

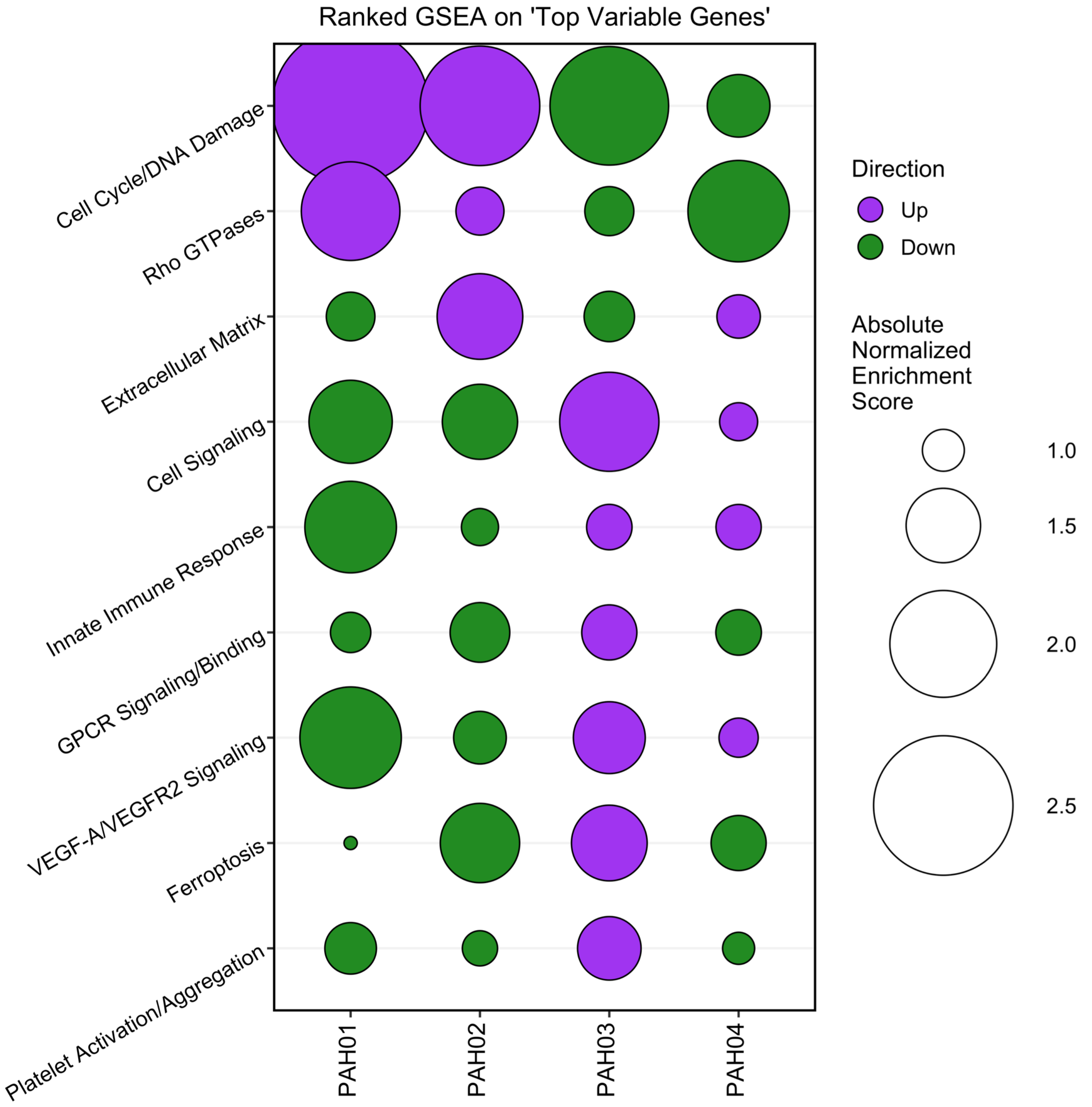

Figure S11: Alternate approach to analysis of the "top variable genes". Ranked gene set enrichment analysis (GSEA) on unclustered "top variable genes" was performed for each patient, with ranking based on descending PAH log_2CPM-based z-scores. All pathways significant in at least one patient were included, using the CP: Canonical Pathways gene sets. Bubble size represents the average absolute normalized enrichment score of pathways grouped by a shared biological theme. Figure generated using the *ggplot2 3.5.1* R package.

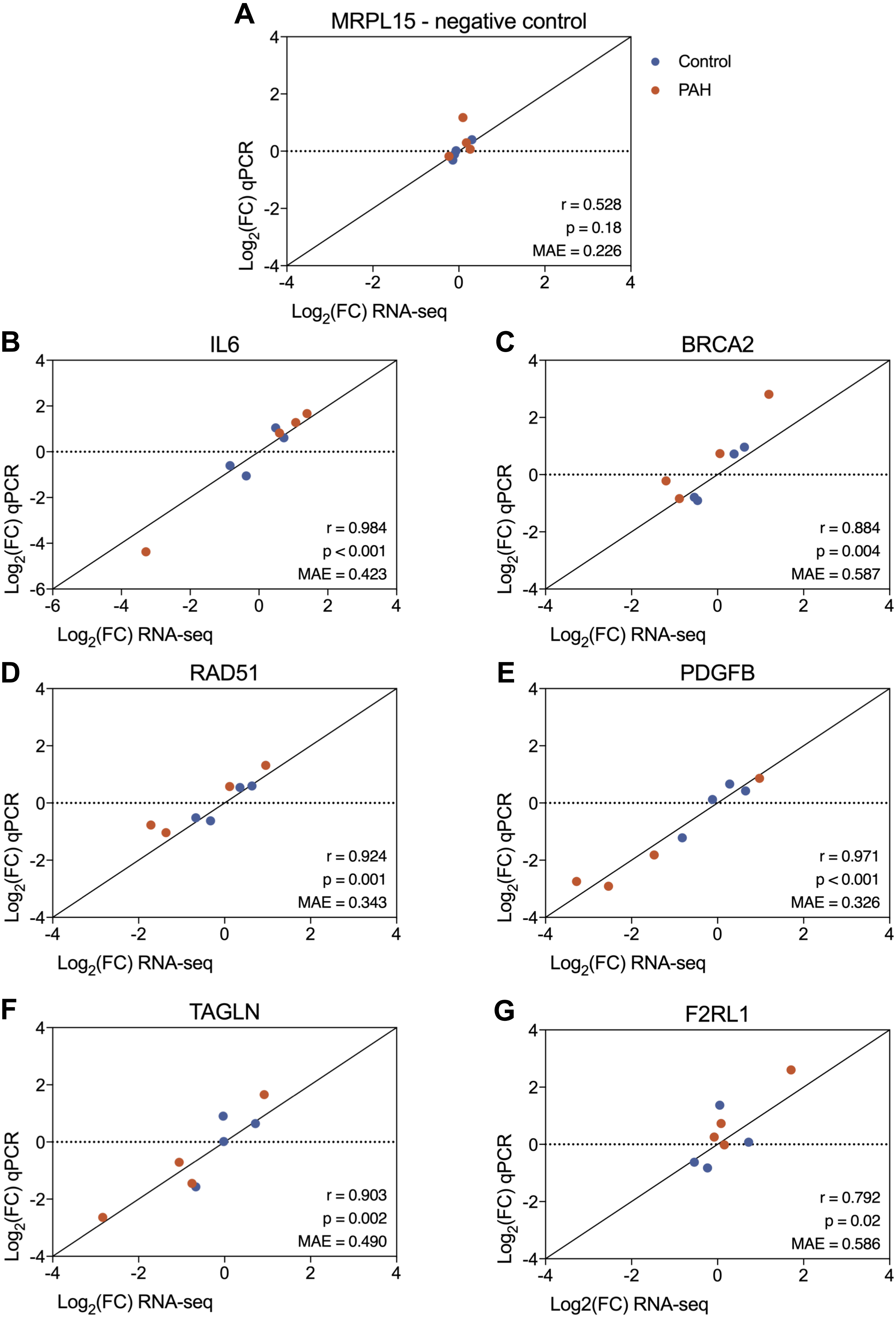

Figure S12: Validation of RNA-seq HSS $\log_2$FC with qPCR in select genes. (A) *MRPL15* as a non-shear-responsive negative control, **(B)** *IL6* as an oppositely-directed ΔΔCV gene, and **(C-G)** *BRCA2*, *RAD51*, *PDGFB*, *TAGLN*, and *F2RL1* as representative genes from the identified cluster pathways all demonstrate strong linear correlation (Pearson r) and low discrepancy (MAE) between methods to quantify control HSS *vs.* PAH HSS $\log_2$FC.
